# Antimicrobial use in lactating sows, piglets, nursery, and grower-finisher pigs on swine farms in Ontario, Canada during 2017 and 2018

**DOI:** 10.1186/s40813-022-00259-w

**Published:** 2022-04-28

**Authors:** Angelina L. Bosman, Anne E. Deckert, Carolee A. Carson, Zvonimir Poljak, Richard J. Reid-Smith, Scott A. McEwen

**Affiliations:** 1 Population Medicine, Ontario Veterinary College, University of Guelph, 50 Stone Road East, Guelph, ON N1G 2W1 Canada; 2 Foodborne Disease and Antimicrobial Resistance Surveillance Division, Centre for Foodborne, Environmental and Zoonotic Infectious Diseases, Infectious Disease Prevention and Control Branch, Public Health Agency of Canada, 370 Speedvale Avenue West, Suite #201, Guelph, ON N1H 7M7 Canada

**Keywords:** Anti-bacterial agent, Antibiotic use, Defined daily dose, Metric, Disease prevention, Disease treatment, Growth promotion, Farm animal, CIPARS, Antimicrobial stewardship

## Abstract

**Background:**

Data on antimicrobial use (AMU) in pig production are needed for the development of good antimicrobial stewardship practices to reduce the risk of antimicrobial resistance in bacteria that can cause illness in animals and humans. In Canada, there is a lack of quantitative data on AMU in the farrowing and nursery stages of pig production. This study aimed to determine which antimicrobial active ingredients are currently used in farrowing, nursery, and grower-finisher herds in the province of Ontario, Canada, and to quantify AMU using various metrics. We collected data on herd demographics, biosecurity, health status, and AMU during one production cycle from 25 farrowing and 25 nursery herds in Ontario, between May 2017 and April 2018, and obtained data from 23 Ontario grower-finisher herds during the same time frame from the Public Health Agency’s Canadian Integrated Program for Antimicrobial Resistance Surveillance. We applied frequency measures, and weight-, and dose-based metrics to the data.

**Results:**

In all pigs, the highest quantity of AMU was administered in-feed. By all routes of administration and compared to other production stages, nursery pigs used more antimicrobials in mg/kg biomass and the number of Canadian defined daily doses per 1000 pig-days (dose_CA_ rate), while grower-finisher pigs used more antimicrobials in total kilograms and the number of Canadian defined daily doses per pig. In suckling pigs in some herds, there was routine disease prevention use of ceftiofur, an antimicrobial active ingredient categorized as very highly important in human medicine by Health Canada. The top antimicrobial used in each stage of pig production often varied by the metric used. There was producer-reported growth promotion use of antimicrobials in suckling and grower-finisher feed.

**Conclusions:**

The results of this study provide a current picture of AMU in pigs in Ontario and can be used as a basis for further research on AMU in farrowing and nursery herds in Canada. Our findings confirm that it would be useful to include farrowing and nursery herds in routine AMU surveillance in Canada. A future analysis using data from this project will examine factors that affect the quantity of AMU.

**Supplementary Information:**

The online version contains supplementary material available at 10.1186/s40813-022-00259-w.

## Background

Antimicrobials play an important role in disease prevention and disease treatment in swine production. The use of antimicrobials in food-animals is linked to the development of antimicrobial resistance in enteric bacteria [1]. Data on antimicrobial use (AMU) is required for the development and evaluation of good antimicrobial stewardship practices, which are vital for reducing the risk of antimicrobial resistance [2].

The Public Health Agency of Canada’s Canadian Integrated Program for Antimicrobial Resistance Surveillance (CIPARS) has been collecting yearly data on AMU in grower-finisher swine herds across Canada since 2006, as part of the CIPARS Farm Swine Surveillance component [3, 4]. Prior to 2006, several studies collected data on AMU in pigs from farrowing to finishing in the province of Ontario [5–7], but no recent studies have examined the use of antimicrobials during the earlier stages of pig production in this province. Ontario is one of the top three pork-producing provinces in Canada [8].

In pigs, antimicrobials are primarily administered to groups of pigs through feed. Antimicrobials can also be administered to groups of pigs in water. Individual animal treatments are more commonly given by injection. Some antimicrobial products are available in Canada for individual oral treatment of pigs; however, it is not known how often they are used in pig production. Some herds in Canada follow a “raised without antimicrobials” program (RWA) where antimicrobials can only be used to treat individual sick pigs and are not permitted to be used in groups of pigs for growth promotion or disease prevention. In RWA herds, sick animals are either humanely euthanized or treated with antimicrobials and removed to a separate facility or stream.

Antimicrobials can be used in healthy pigs for disease prevention or growth promotion, or in clinically ill pigs for disease treatment. Many countries including Canada no longer permit the use of medically important antimicrobials for growth promotion [9–11], although at the time of this study (May 2017 through April 2018), growth promotion use was still permitted in Canada (ceased December, 2018). Antimicrobials are categorized by Health Canada’s Veterinary Drugs Directorate according to their importance in human medicine. Categories include I (very high importance), II (high importance), III (medium importance), and IV (low importance) [12]. Ionophores are considered antimicrobials for regulatory purposes in Canada and are included in category IV. Other antimicrobial categorization systems include those developed by the European Medicines Agency (EMA) [13], the World Health Organization (WHO) [14], and the World Health Organisation for Animal (OIE) [15]. Health Canada, EMA, and the WHO categorize antimicrobials according to their importance in human medicine, while the OIE categorizes antimicrobials according to their importance in veterinary medicine. Health Canada’s Category I classification most closely matches EMA categories “Avoid” (e.g., carbapenems, glycopeptides) and “Restrict” (e.g., 3^rd^ generation cephalosporins, fluoroquinolones), including antimicrobials that have limited or no alternatives in human medicine in case of resistance. Streptogramins, classified by Health Canada as Category II, are included in EMA’s highest category “Avoid”.

There are many antimicrobial use metrics which can describe the frequency and quantity of antimicrobials used [16, 17]. These metrics can be frequency-based, weight-based, or dose-based [18]. Frequency-based metrics are simple and intuitive. Weight-based metrics provide more information than frequency-based metrics but require more data to produce. Dose-based metrics have the advantage of adjusting for the daily dose of the antimicrobial, which facilitates comparisons in use when different antimicrobials are used [19]. Dose-based metrics require the use of standard drug doses, which were assigned in 2019 for all antimicrobials labelled for use in pigs in Canada [20].

This study aimed to determine which antimicrobial active ingredients are currently used in farrowing (suckling pigs and lactating sows), nursery, and grower-finisher herds in the province of Ontario, Canada. It also aimed to estimate the frequency and quantity of AMU in each production stage using frequency measures, and weight, and dose-based metrics. We anticipated that larger quantities of antimicrobials would be used in the nursery stage of production compared to the farrowing or grower-finisher stages.

## Methods

We obtained data on Ontario grower-finisher herds sampled between May 2017 and April 2018 from CIPARS. If a grower-finisher herd was sampled twice during this time period (once in 2017 and once in 2018), the 2017 sample was removed, to try to achieve an even distribution of samples over the 12-month period. Data from all 23 available grower-finisher herds were included in the study. The methods used to collect AMU data in grower-finisher pigs are well described in the CIPARS 2017 Design and Methods report [4]. Briefly, herd veterinarians from every veterinary practice in Ontario that specialized in pigs were contracted to recruit producers from a representative sample of grower-finisher herds in their practice. Herds on an RWA program could be included as long as the proportion of RWA herds in the sample reflected the proportion of RWA herds in the practice. These veterinarians completed a questionnaire by interviewing the producer on AMU, herd demographics, biosecurity, and herd health, during the most recently completed production cycle. Herd health data included the status of the herd as reported by the producer with regards to a list of major swine diseases and infections. Options for reporting health status included “confirmed positive” or “confirmed negative” (with laboratory testing), “likely positive” or “likely negative” (with no laboratory testing), and “unknown”. The vaccination status of the pigs for each disease/agent and whether antimicrobials were used to treat or prevent each disease/agent were also requested. Animals in the herd were not handled or sampled during the course of the study; data was collected through interview only with the producer.

We used the same methodology to collect data from 25 sow and 25 nursery herds in Ontario during the same period (May 2017 to April 2018), with modifications as needed to tailor the methods to each production stage. Modifications included adjusting the questionnaires to account for the different ways that antimicrobials were used during the farrowing and nursery periods (Table 1). The farrowing questionnaire was modified to collect information on lactating sows and suckling pigs (Additional file 1). Information was collected on the total number of sows at the site and the total nursery pig capacity of the barn was requested for the nursery operations (Additional file 2). Additional information that was unique to the farrowing and nursery questionnaires included the estimated percentage of creep feed wastage, the average age at weaning, minimum, maximum and average sow parity, and extra-physiologic additions of zinc to nursery pig feed. Feed wastage was only estimated for creep feed as wastage during this first introduction to solid food can be considerable, depending on the design of the farrowing crates/pens, and our goal was to obtain an estimate of antimicrobial exposure for future investigations into the relationship between antimicrobial exposure and antimicrobial resistance.

**Table 1:** Data collected by questionnaire on use of antimicrobial active ingredients (AAI) specific to each route of administration and type of pig

Route/means of administration	Questionnaire(s)^a^	Information collected
Creep feed	Farrowing^b^	AAI name, grams of AAI per tonne feed, primary reason for use, age at start and end, % of piglets fed, % creep feed wasted
Oral—individual	Farrowing^b^	AAI name and concentration, product name, volume given, primary reason for use, duration of use, weight and age at start of treatment, % pigs exposed
Regular feed	Farrowing, nursery, grower-finisher	Ration name, medicated or unmedicated, AAI name, grams AAI per tonne feed, primary reason for use, start and end weight of pigs, duration fed, % pigs fed
Water	Farrowing, nursery, grower-finisher	AAI name and concentration, product name, grams per liter water, primary reason for use, duration of use, weight and age at start of treatment, % pigs exposed
Injection	Farrowing, nursery, grower-finisher	AAI name and concentration, product name, volume administered per pig per day, primary reason for use, duration of use, weight and age at start of treatment, % pigs exposed

*AAI* antimicrobial active ingredient

^a^The farrowing questionnaire collected data on lactating sows and suckling pigs

^b^Suckling pigs only

**Fig. 1:**
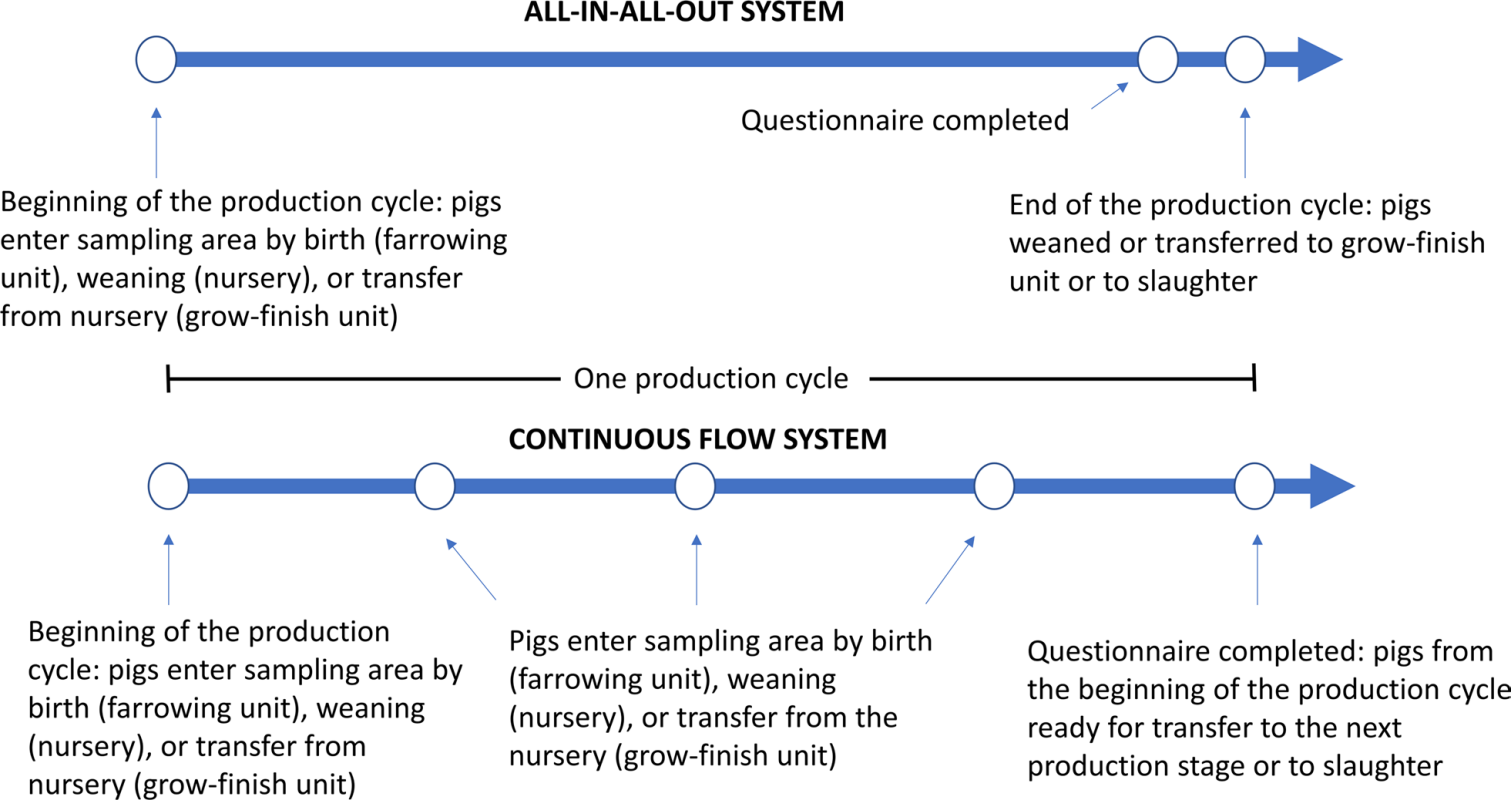
Flow chart illustrating the data collection period of one production cycle for all-in-all-out and continuous flow production systems in participating sow, nursery, and grower-finisher pig herds in the province of Ontario, Canada

The types of AMU information collected for each route of administration in the sow, nursery, and grower-finisher questionnaires are described in Table 1. At each farm, AMU data were collected for the current production cycle from a batch of pigs that were close to transferring to the nursery (sows and suckling pigs), to the grower-finisher unit (nursery pigs), or to slaughter (grower-finisher pigs). The sampling area included the room(s) or pen(s) in which the batch of pigs were housed, and the data collection period was the duration of the current production cycle. For all-in-all-out systems, the end of the data collection period was the anticipated end of the current production cycle, and the beginning of the data collection period was the day the first pigs entered the sampling area during the current production cycle (Fig. 1). For continuous flow systems, the end of the data collection period was the day the questionnaire was completed, and the beginning of the data collection period was calculated as the day the questionnaire was completed minus the average length of the production cycle (Fig. 1). The number of pigs at risk of exposure to antimicrobials was calculated by subtracting half the mortality from either the number of pigs at the start of the production cycle (all-in-all-out systems) or the number of pigs that entered the sampling area during the data collection period (continuous flow systems).

### Data entry, validation, and analysis

Each completed farrowing and nursery questionnaire was reviewed for unanswered questions and nonsensical entries, and if found, the herd veterinarian or representative who completed the questionnaire was contacted to obtain the missing information and/or clarify entries where possible. Data were entered manually into a Post-SQL database.

For ease of analysis, we adopted the CIPARS Farm Surveillance programs methodology of assigning a positive disease status to herds that reported a “confirmed positive” or “likely positive” status for a particular disease or disease agent, and a negative disease status to herds that reported a “confirmed negative” or “likely negative” status [4]. While laboratory confirmation of disease is always preferred, it is not always performed, for various reasons, and if a veterinarian or producer feels the herd is affected by a particular disease or disease agent, then this may influence their AMU decisions, regardless of whether lab confirmation is performed.

To estimate the amount of antimicrobial consumed in feed, it was necessary to estimate the amount of feed consumed by the pigs at risk. Feed consumption was calculated within the database using the following methodologies. For sows, the National Research Council estimate of 5966 g feed/day was used (6280 g/day minus 5% wastage) [21]. For suckling pigs, the amount of creep feed consumed was estimated using a regression curve obtained from Sulabo et al. [22]. For each herd, the age of the piglets when creep feed was initially started, and the weaning age of the pigs were used to determine the amount of feed consumed by a standardized litter of 11 pigs. The result was divided by 11 to obtain the amount of creep feed consumed per piglet, then multiplied by the number of piglets at risk of exposure to antimicrobials (calculated as the number of piglets born alive during the data collection period less half the mortality). The percentage of creep feed wastage was estimated by the producer and applied against the calculated amount of creep feed consumed by the sampled piglets.

For nursery pigs, feed consumption was determined using the same methods as those used to calculate feed consumption for grower-finisher pigs, as described in the CIPARS 2017 Design and Methods [4]. Briefly, the average daily gain (ADG) of each herd was calculated using data from the questionnaire. For nursery pigs, instead of National Research Council tables used for grower-finisher pigs, a feed calculator developed by Kansas State University was used to generate three plots of kilograms of feed consumption per day for each ration fed. One curve was generated for herds with poor performance (25th percentile for ADG), one for high performance herds (75th percentile for ADG), and one for herds with average performance (between the 25th and 75th percentile for ADG). Like the methodology used for grower-finisher pigs, simple regression and integral calculus were used to determine the cumulative feed consumption per pig, which was then multiplied by the number of pigs at risk.

As with feed, it was necessary to determine how much water the pigs drank in order to estimate the quantity of antimicrobials consumed in water. For lactating sows, the NRC estimate of 18 L/day in lactating sows was used [21]. For nursery pigs, water intake was estimated using Eq. 1, obtained from Brooks et al. [23].

1 $$Water\, intake\, \left( {L/day} \right) = 0.149 + \left( {3.053*mean\,daily\,feed\,intake\, \left( {kg/day} \right)} \right)$$

The total grams of antimicrobial consumed was then determined by multiplying the water intake (L/day) with the dose of antimicrobial (g/L) and the duration of use (days).

A descriptive analysis was performed using R 3.6.3 [24], and package dplyr [25]. The quantity of antimicrobial consumed in feed, in water, and by injection was described using frequency measures and weight- and dose-based metrics. Frequency measures included the percentage of farms using antimicrobials by route of administration. Weight-based metrics included the milligrams of antimicrobial consumed adjusted by the kilograms of biomass (mg/kg biomass), where biomass is the number of animals at risk of treatment with antimicrobials multiplied by the average weight at treatment. Dose-based metrics included the number of Canadian defined daily doses for animals per pig (nDDDvetCA/pig) and the number of Canadian defined daily doses for animals per 1000 pig-days (nDDDvetCA/1000 pig-days) [26]. We used the European Medicine Agency’s average weight at treatment for suckling pigs (4 kg), grower-finisher pigs (65 kg), and sows (240 kg) [19, 27]. Since the European Medicine Agency does not report an average weight at treatment for nursery pigs, one was calculated from the data in this study (11.5 kg). A treatment was defined as a continuous daily administration of one (or more) antimicrobials at a specific dose. If the dose changed or if administration was paused and then restarted, it was considered a new treatment.

## Results

**Fig. 2:**
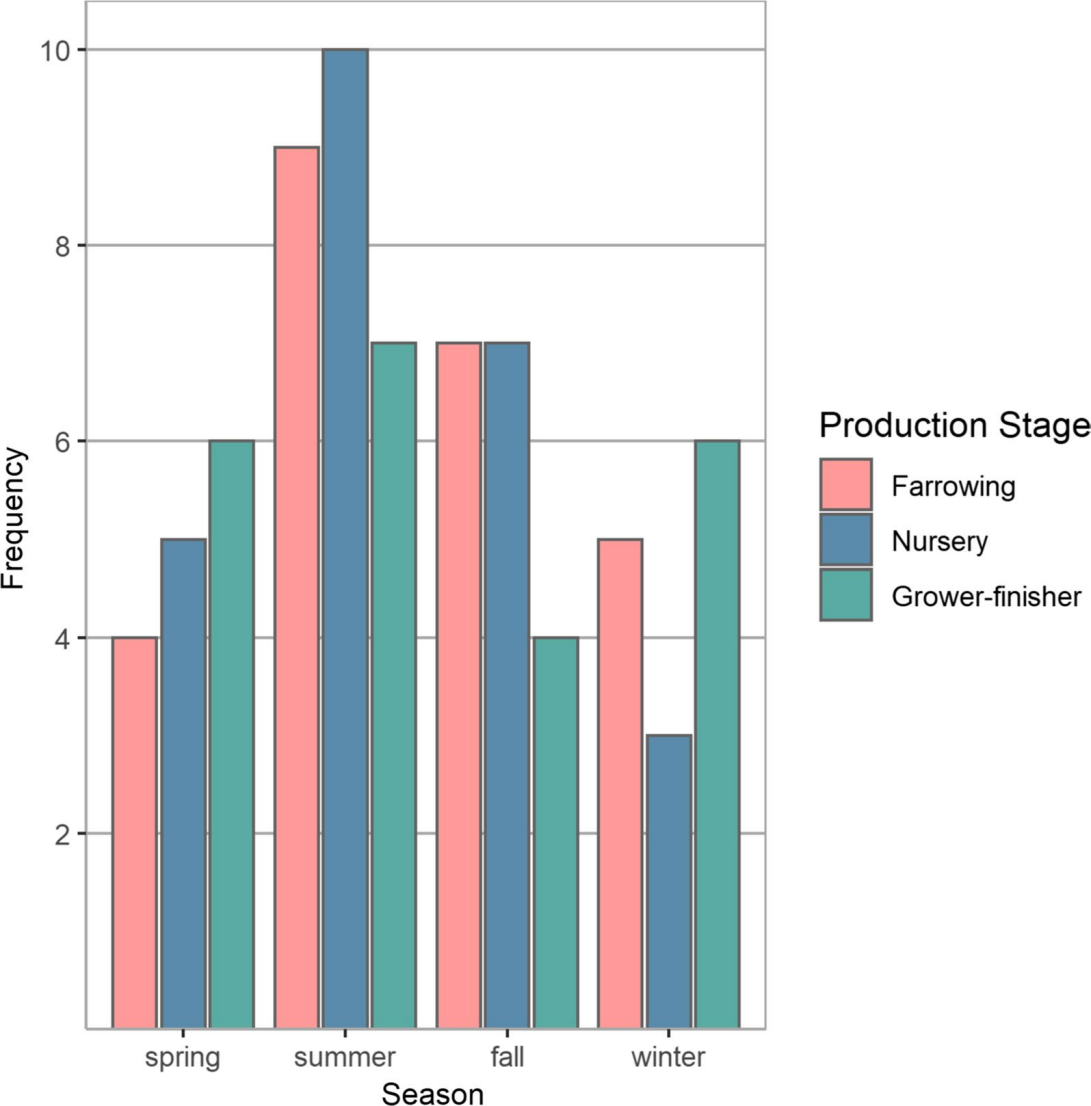
The number of herds by season in which data collection and sampling took place (n = 25 nursery, 25 sow herds, 23 grower-finisher herds, May 2017–April 2018). spring = March–May, summer = June–August, fall = September–November, winter = December–February

Common ownership/management linked twenty-four of the 25 sow herds to 24/25 of the nursery herds and 8/23 grower-finisher herds. Data collection was distributed throughout the 12-month period from May to April, with the majority occurring in the summer and fall months (Fig. 2).

The number of sows per sow herd ranged from 100 to 1600. The nursery pig capacity of the herds ranged from 400 to 3200 pigs, and the grower-finisher capacity ranged from 750 to 2400 pigs. Most herds were managed all-in-all-out, with four sow, four nursery, and four grower-finisher continuous flow herds. Most sow and nursery herds were independent (21 and 15 herds, respectively) versus part of a production group (4 and 10 herds, respectively), while most grower-finisher herds were part of a production group (15 herds). Four sow herds, two nursery herds and one grower-finisher herd were on a “raised without antibiotics” program, where antimicrobials were not used for growth promotion or disease prevention purposes but were used when needed for individual treatment of clinically ill animals [28]. The length of the production cycle, equivalent to the time at risk of treatment with antimicrobials, was skewed slightly to the right for each production stage by a small number of outlier herds (Table 2).

**Table 2:** Descriptive statistics of length of the production cycle (time at risk of treatment with antimicrobials) by production stage based on start and end dates provided by the participants

Production stage	Minimum days	Maximum days	Mean days	Median days
Farrowing	18	41	25	23
Nursery	28	99	56	52
Grower-finisher	87	157	114	112

### Biosecurity

**Fig. 3:**
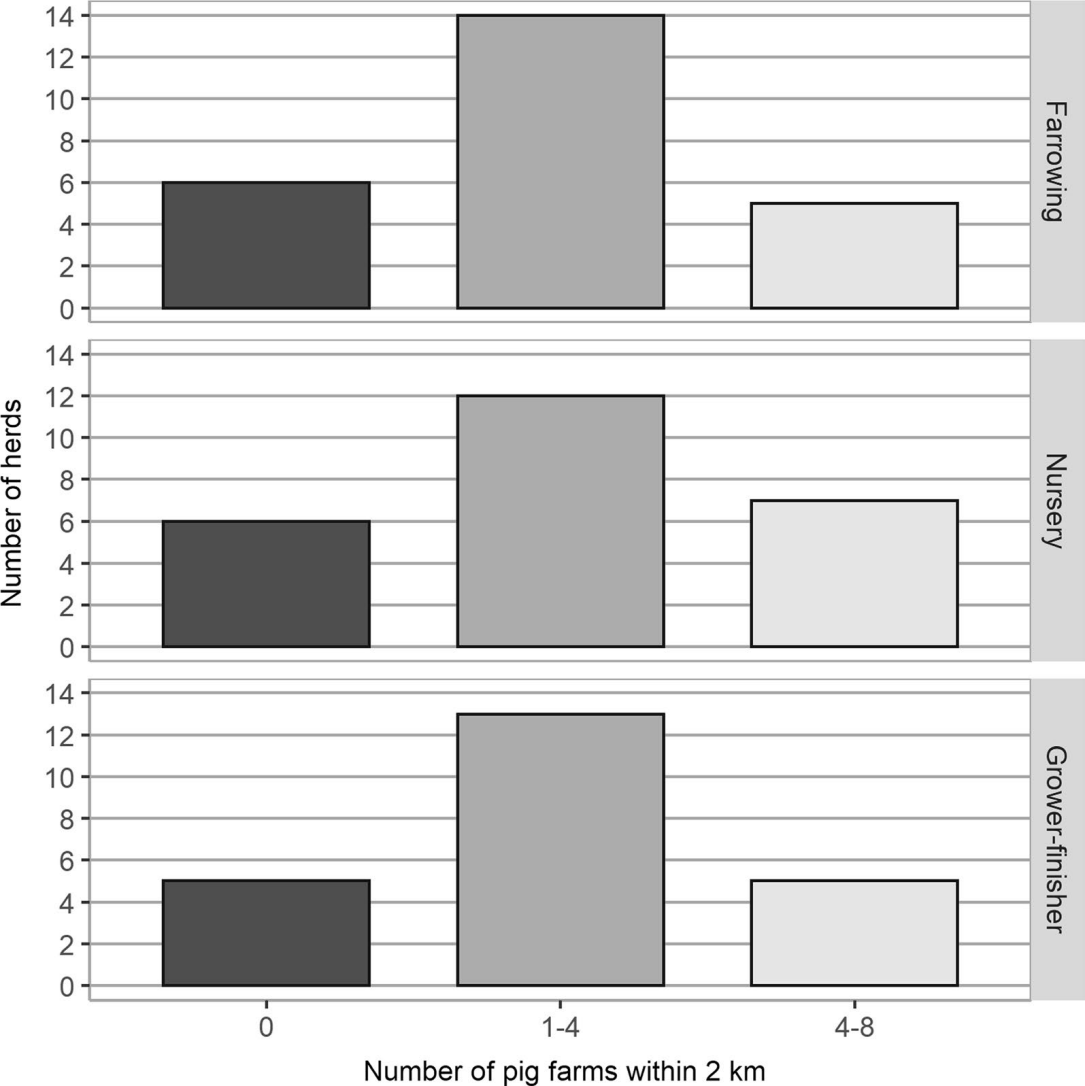
The number of farrowing, nursery, and grower-finisher herds by the number of swine farms within 2 km of the operation (n = 25 farrowing, 25 nursery, 23 grower-finisher herds, May 2017–April 2018)

The frequency of use of various biosecurity practices in the study herds was high, except for the use of boot dips and the quarantine of new gilts in sow herds (Table 3). No additional biosecurity practices were identified by the participants beyond those listed in Table 3. The average number of downtime hours required for visitors and personnel after contact with other pigs or swine farms was 32 h for all three herd types, among herds with required downtime. The number of swine farms within two km ranged from zero to eight (Fig. 3).

**Table 3:** Biosecurity practices by herd type for 25 nursery, 25 farrowing, and 23 grower-finisher herds in Ontario, Canada, May 2017–April 2018

Biosecurity practice	Participating herds using practice											
	Sow herds		95% confidence interval		Nursery herds		95% confidence interval		Grower-finisher herds		95% confidence interval	
	n	(%)			n	(%)			n	(%)		
Barn boots	25	100	0.86	1.00	25	100	0.86	1.00	23	100	0.85	1.00
Coveralls	24	96	0.80	1.00	24	96	0.80	1.00	23	100	0.85	1.00
Boot dip	3	12	0.03	0.31	5	20	0.07	0.41	3	13	0.03	0.34
Biosecurity sign	25	100	0.86	1.00	25	100	0.86	1.00	23	100	0.85	1.00
Danish entry^a^	23	92	0.14	0.99	23	92	0.74	0.99	20	87	0.66	0.97
Barn locked	19	76	0.55	0.91	21	84	0.64	0.95	18	78	0.56	0.93
Visitors restricted	25	100	0.86	1.00	24	96	0.80	1.00	23	100	0.85	1.00
Shower in	19	76	0.55	0.91	18	72	0.51	0.88	13	57	0.35	0.77
Quarantine new gilts	10	40	0.21	0.61	NA	NA	NA	NA	NA	NA	NA	NA
Downtime^b^	21	84	0.64	0.95	21	84	0.64	0.95	21	91	0.72	0.99

^a^A biosecurity tool that uses a solid partition to divide the entryway of the barn into “clean” and “dirty” areas with storage for clothing and boots on both sides

^b^Downtime refers to the requirement for visitors and personnel to refrain from visiting the farm for a certain length of time after contact with other swine farms

### Health status

Farrowing herds were most often reported positive for *Escherichia coli* (*E. coli*), erysipelas, *Streptococcus suis*, and porcine circovirus associated disease (PCVAD) (Table 4). Nursery herds were most often reported positive for *Streptococcus suis*, PCVAD, *Haemophilus parasuis*, and *E. coli* (Table 5). Positive status was most often reported for *Streptococcus suis*, PCVAD, ileitis (*Lawsonia* spp*.*), and *E. coli* in grower-finisher herds (Table 5). Most herds were reportedly negative for *Actinobacillus pleuropneumoniae,* porcine epidemic diarrhea, and porcine reproductive and respiratory syndrome.

**Table 4:** Suckling pig and sow disease status of herds as reported by producers for 25 sow herds in the province of Ontario, Canada (May 2017–April 2018)

Disease/agent	Sows						Suckling pigs					
	Positive^a^ n (%)		Negative^a^ n (%)		Don’t know n (%)		Positive^a^ n (%)		Negative^a^ n (%)		Don’t know n (%)	
APP	1	(4)	21	(84)	3	(12)	1	(4)	23	(92)	1	(4)
*Escherichia coli*	23	(92)	2	(8)	0	(0)	23	(92)	2	(8)	0	(0)
Erysipelas	23	(92)	2	(8)	0	(0)	15	(60)	9	(36)	1	(4)
*Haemophilus parasuis*^b^	17	(68)	6	(24)	2	(8)	15	(60)	6	(24)	4	(16)
Ileitis	17	(68)	5	(20)	3	(12)	11	(44)	8	(32)	6	(24)
Influenza	15	(60)	7	(28)	3	(12)	13	(52)	8	(32)	4	(16)
Mycoplasma	14	(56)	10	(40)	1	(4)	11	(44)	14	(56)	0	(0)
PCVAD	19	(76)	5	(20)	1	(4)	19	(76)	6	(24)	0	(0)
PED	0	(0)	24	(96)	1	(4)	1	(4)	23	(92)	1	(4)
PRRS	9	(36)	16	(64)	0	(0)	5	(20)	19	(76)	1	(4)
*Salmonella* spp*.*	4	(16)	10	(40)	11	(44)	5	(20)	14	(56)	6	(24)
*Streptococcus suis*	20	(80)	3	(12)	2	(8)	20	(80)	2	(8)	3	(12)

*APP* actinobacillus pleuropneumoniae, *PCVAD* porcine circovirus associated disease, *PED* porcine epidemic diarrhea, *PRRS* porcine respiratory and reproductive syndrome

^a^Herds were classified positive if the questionnaire response was “confirmed positive” or “likely positive”; Herds were classified negative if the questionnaire response was “confirmed negative” or “likely negative”

^b^*Haemophilus parasuis* is also known as *Glaesserella parasuis*

**Table 5:** Grower-finisher and nursery pig disease status of herds as reported by producers for 23 grower-finisher and 25 nursery herds in the province of Ontario, Canada (May 2017–April 2018)

Disease/agent	Nursery pigs						Grower-finisher pigs					
	Positive^a^ n (%)		Negative^a^ n (%)		Don’t know n (%)		Positive^a^ n (%)		Negative^a^ n (%)		Don’t know n (%)	
APP	1	(4)	23	(92)	1	(4)	2	(9)	20	(87)	1	(4)
*Escherichia coli*	19	(76)	4	(16)	2	(8)	2	(9)	20	(87)	1	(4)
Erysipelas	16	(64)	7	(28)	2	(8)	16	(70)	6	(26)	1	(4)
*Haemophilus parasuis*^b^	20	(80)	2	(8)	3	(12)	17	(74)	3	(13)	3	(13)
Ileitis	14	(56)	7	(28)	4	(16)	18	(78)	4	(17)	1	(4)
Influenza	16	(64)	5	(20)	4	(16)	16	(70)	5	(22)	2	(9)
Mycoplasma	14	(56)	10	(40)	1	(4)	15	(65)	8	(35)	0	(0)
PCVAD	20	(80)	5	(20)	0	(0)	20	(87)	3	(13)	0	(0)
PED	0	(0)	24	(96)	1	(4)	1	(4)	20	(87)	2	(9)
PRRS	8	(32)	17	(68)	0	(0)	11	(48)	12	(52)	0	(0)
*Salmonella* spp*.*	5	(20)	14	(56)	6	(24)	11	(48)	6	(26)	6	(26)
*Streptococcus suis*	24	(96)	0	(0)	1	(4)	20	(87)	1	(4)	2	(9)
TGE	0	(0)	22	(88)	3	(12)	0	(0)	22	(96)	1	(4)

*APP* actinobacillus pleuropneumoniae, *PCVAD* porcine circovirus associated disease, *PED* porcine epidemic diarrhea, *PRRS* porcine respiratory and reproductive syndrome, *TGE* transmissible gastroenteritis

^a^Herds were classified positive if the questionnaire response was “confirmed positive” or “likely positive”; Herds were classified negative if the questionnaire response was “confirmed negative” or “likely negative”

^b^*Haemophilus parasuis* is also known as *Glaesserella parasuis*

Suckling, nursery, and grower-finisher pigs were most commonly vaccinated against porcine circovirus type 2 for the prevention of PCVAD (Table 6), while sows were most commonly vaccinated against erysipelas and *E. coli*. Sows were vaccinated against more pathogens than suckling, nursery, or grower-finisher pigs.

**Table 6:** The number and percentage of herds that were vaccinated against common swine disease conditions, by type of pig for 25 nursery, 25 farrowing, and 23 grower-finisher herds in the province of Ontario, Canada (May 2017–April 2018)

Disease/agent	Suckling pigs		Sows		Nursery pigs		Grower-finisher pigs	
	n	(%)	n	(%)	n	(%)	n	(%)
APP	0	(0)	0	(0)	0	(0)	0	(0)
*Escherichia coli*	0	(0)	21	(84)	1	(4)	0	(0)
Erysipelas	1	(4)	25	(100)	1	(4)	2	(9)
*Haemophilus parasuis*	2	(8)	11	(44)	3	(12)	0	(0)
Ileitis	8	(32)	14	(56)	10	(40)	2	(9)
Influenza	0	(0)	3	(12)	0	(0)	0	(0)
Mycoplasma	11	(44)	11	(44)	17	(68)	7	(30)
PCVAD	17	(68)	12	(48)	22	(88)	8	(35)
PED	0	(0)	0	(0)	0	(0)	0	(0)
PRRS	0	(0)	5	(20)	0	(0)	1	(4)
*Streptococcus suis*	0	(0)	2	(8)	0	(0)	0	(0)
*Salmonella* spp*.*	NA	NA	NA	NA	1	(4)	0	(0)
TGE	NA	NA	NA	NA	0	(0)	0	(0)

*APP* actinobacillus pleuropneumoniae, *PCVAD* porcine circovirus associated disease, *PED* porcine epidemic diarrhea, *PRRS* porcine respiratory and reproductive syndrome, *TGE* transmissible gastroenteritis, *NA* not applicable

The use of antimicrobials was most commonly reported for treating or controlling *E. coli* and *Streptococcus suis* in suckling pigs, erysipelas and *Haemophilus parasuis* in sows, *Streptococcus suis*, *Haemophilus parasuis* and *E. coli* in nursery pigs, and ileitis (*Lawsonia* spp*.*) and mycoplasma in grower-finisher pigs (Table 7). Antimicrobials were used against a broader range of diseases in nursery pigs than in other types of pigs.

**Table 7:** The number of herds reporting the use of antimicrobials for treatment or control of common swine diseases, by type of pig (May 2017–April 2018)

Disease/agent	Suckling pigs		Sows		Nursery pigs		Grower-finisher pigs	
	n	(%)	n	(%)	n	(%)	n	(%)
APP	0	(0)	0	(0)	0	(0)	0	(0)
*Escherichia coli*	13	(52)	2	(8)	10	(40)	2	(9)
Erysipelas	1	(4)	5	(20)	1	(4)	0	(0)
*Haemophilus parasuis*	8	(32)	4	(16)	11	(44)	3	(13)
Ileitis	1	(4)	2	(8)	5	(20)	8	(35)
Influenza	0	(0)	1	(4)	1	(4)	0	(0)
Mycoplasma	1	(4)	2	(8)	7	(28)	8	(35)
PCVAD	1	(4)	0	(0)	1	(4)	0	(0)
PED	0	(0)	0	(0)	0	(0)	0	(0)
PRRS	0	(0)	1	(4)	3	(12)	1	(4)
*Salmonella* spp*.*	0	(0)	0	(0)	1	(4)	1	(4)
*Streptococcus suis*	10	(40)	2	(8)	15	(60)	5	n
TGE	NA	NA	NA	NA	0	0	0	0

*APP* actinobacillus pleuropneumoniae, *PCVAD* porcine circovirus associated disease, *PED* porcine epidemic diarrhea, *PRRS* porcine respiratory and reproductive syndrome, *TGE* transmissible gastroenteritis, *NA* not applicable

### Antimicrobial use

The percentage of farms using antimicrobials by any route of administration during the sampled production stage was 92% in suckling pigs, 92% in sows, 96% in nursery pigs, and 70% in grower-finisher pigs. The percentage of farms using antimicrobials in sow feed was 24%, in nursery feed 76% and in grower-finisher feed 65%. Sixty percent of sow herds offered creep feed, all of which was medicated with antimicrobials. More herds used antimicrobials by injection in sows (84%) and suckling pigs (84%), than nursery pigs (60%) and grower-finishers (35%). Antimicrobials were only used in water by nursery herds (40%) and grower-finisher herds (13%). All four of the sow herds on an RWA program and one of the two RWA nursery herds used antimicrobials by injection for disease treatment purposes. The other RWA nursery herd and the one RWA grower-finisher herd did not use any antimicrobials during the sampled production cycle by any route of administration.

The antimicrobial active ingredient (AAI) used by the highest percentage of farms in sow feed, creep feed, and nursery feed was chlortetracycline (12%, 56%, and 72%, respectively). Lincomycin was used by the highest percentage of farms in grower-finisher feed (43%). By injection, procaine penicillin G was used by the highest percentage of farms in sows, suckling pigs, nursery pigs, and grower-finisher pigs (52%, 56%, 52%, and 26%, respectively). In water, amoxicillin was used by the highest percentage of nursery farms (20%), and penicillin G potassium was used by the highest percentage of grower-finisher farms (9%).

The quantity of antimicrobials used in feed, as measured using weight and dose-based metrics, was higher than the quantity used in water or by injection in all types of pigs (Table 8). Nursery pigs used the most antimicrobials in feed as measured in mg/kg biomass and the dose_CA_ rate, while grower-finishers used the most antimicrobials in feed as measured in total kilograms and defined daily doses per pig. By injection, suckling pigs used the most antimicrobials as measured in mg/kg biomass, doses_CA_ per pig, and the dose_CA_ rate. More antimicrobials were used in water in nursery pigs than in grower-finisher pigs as measured by all three quantitative metrics.

**Table 8:** The quantities of antimicrobials used in sows, suckling pigs, nursery pigs, and grower-finisher pigs by route of administration, including the percentage of herds, number of pigs at risk and the number of pigs exposed (25 sow, 25 nursery, 23 grower-finisher herds, May 2017–April 2018)

ROA	Percentage of herds (%)	Pigs at risk	Total kg^a^	Mg/kg biomass	Doses_CA_/pig^b^	Dose_CA_ rate^b^
*Sows*						
Feed	24	781	9332	49.8	6.8	297.6
Injection	84	781	1895	10.1	1.4	61.1
All routes	92	781	11,227	59.9	8.2	358.7
*Suckling pigs*						
Feed	60	9994	1516	37.9	5.8	252.3
Injection	84	9994	843	21.1	5.1	220.6
All routes	92	9994	2359	59.0	10.9	472.9
*Nursery pigs*						
Feed	76	13,251	32,525	213.4	24.1	463.4
Injection	60	13,251	275	1.8	0.2	4.1
Water	40	13,251	9354	61.4	3.6	69.2
All routes	96	13,251	42,155	274.2	27.9	536.8
*Grower-finisher pigs*						
Feed	65	21,638	161,760	115.0	29.8	266.1
Injection	35	21,638	487	0.3	0.04	0.4
Water	13	21,638	20,592	14.6	1.0	8.6
All routes	70	21,638	182,839	130.0	30.8	275.1

*ROA* route of administration

^a^Total kg = the total kilograms of antimicrobial used

^b^Doses_CA_ per pig = number of Canadian defined daily doses per pig; Dose_CA_ rate = number of Canadian defined daily doses per 1000 pig-days

#### Lactating sows

The median length of the farrowing period of 23 days (Table 2), was used as the time at risk for calculation of the animal-time denominator. In lactating sows, chlortetracycline, oxytetracycline and procaine penicillin G were used in feed by the highest number of herds. The ranking of AAIs used in feed varied between weight- and dose-based metrics (Table 9). The top AAIs used in feed as measured in mg/kg biomass was chlortetracycline, followed by oxytetracycline and bacitracin. The top AAI used in feed as measured in both dose-based metrics was bacitracin, followed by oxytetracycline and chlortetracycline. All antimicrobial use in feed was for disease prevention, against respiratory or enteric diseases, lameness, urinary tract infections and post-farrowing infections.

**Table 9:** The quantities of antimicrobial active ingredients used in lactating sow feed as measured in weight-based and dose-based metrics (25 sow herds in the province of Ontario, Canada, May 2017–April 2018)

ROA	Antimicrobial active ingredients	Class	Number of herds	Mg/kg biomass	Doses_CA_/pig^a^	Dose_CA_ rate^a^
Feed	Bacitracin	Bacitracins	1	12.25	2.72	118.4
	Chlortetracycline	Tetracyclines	3	19.73	1.90	82.5
	Oxytetracycline	Tetracyclines	2	15.13	1.99	86.6
	Procaine penicillin G	Penicillins	2	3.17	0.24	10.4
	Sulfamethazine	Sulfonamides	1	0.69	0.16	6.8
	Tilmicosin	Macrolides	1	0.31	0.31	13.6
Injection	Benzathine penicillin G (with other antimicrobials)	Penicillins	2	0.36	0.30	12.89
	Ceftiofur	Cephalosporins	1	0.02	0.01	0.31
	Oxytetracycline	Tetracyclines	7	1.50	0.25	11.07
	Procaine penicillin G	Penicillins	10	4.34	0.32	13.97
	Procaine penicillin G LA	Penicillins	3	0.58	0.09	3.76
	Sulfadoxine (with trimethoprim)	Sulfonamides	9	2.76	0.21	9.03
	Trimethoprim (with sulfadoxine)	Dihydrofolate reductase inhibitors	9	0.55	0.23	10.01

*ROA* route of administration

^a^Doses_CA_ per pig = number of Canadian defined daily doses per pig; Dose_CA_ rate = number of Canadian defined daily doses per 1000 pig-days

By injection, procaine penicillin G, trimethoprim sulfadoxine, and oxytetracycline were the top AAIs used as measured by the number of herds and mg/kg biomass (Table 9). In both defined daily dose metrics, the top AAI used by injection was procaine penicillin G followed by benzathine penicillin G and oxytetracycline (Table 9). Seventy-six percent of antimicrobial use by injection was for treatment of diseases (i.e., clinical infections), including respiratory disease, lameness, and post-farrowing infections such as metritis and mastitis. Other reasons for injecting antimicrobials included poor doing, off-feed, and fever. Twenty-four percent of antimicrobial use by injection was for disease prevention, including prevention of enteric disease, lameness, post-farrowing infections, and off-feed.

#### Suckling pigs

As for sows, the median length of the farrowing period of 23 days (Table 2), was used as the time at risk for calculation of the animal-time denominator. The top AAI used in creep feed did not vary by metric, with chlortetracycline having the highest frequency (number of herds) and quantity of use (Table 10). In mg/kg biomass, the next highest AAI was tiamulin, followed by tylosin. In both defined daily dose metrics, the next highest AAI was salinomycin, followed by tiamulin. Seventy-five percent of antimicrobial use in creep feed was to prevent disease, with the remainder for growth promotion or unknown (information not provided). AAIs used in creep feed for growth promotion included chlortetracycline (Health Canada category III—medium importance) and tiamulin (not categorized by Health Canada). There was no reported use of individual oral administration of antimicrobials.

**Table 10:** The quantities of antimicrobial active ingredients used in creep feed for suckling pigs as measured in weight-based and dose-based metrics (25 sow herds in the province of Ontario, Canada, May 2017–April 2018)

ROA	Antimicrobial active ingredient	Class	Number of herds	Mg/kg biomass	Doses_CA_/pig^a^	Dose_CA_ rate^a^
Feed	Chlortetracycline	Tetracyclines	14	27.8	2.67	116.1
	Procaine penicillin G	Penicillins	2	0.5	0.04	1.7
	Salinomycin	Ionophores	1	1.3	1.30	56.4
	Sulfamethazine	Sulfonamides	2	1.0	0.24	10.4
	Tiamulin	Pleuromutilins	12	5.3	0.95	41.2
	Tylosin	Macrolides	1	1.9	0.61	26.6
Injection	Benzathine penicillin G (with other antimicrobials)	Penicillins	3	2.57	2.14	93.08
	Ceftiofur	Cephalosporins	2	0.13	0.04	1.92
	Ceftiofur LA	Cephalosporins	4	0.77	0.77	33.50
	Enrofloxacin	Fluoroquinolones	2	0.10	0.01	0.57
	Gentamicin	Aminoglycosides	1	0.02	0.01	0.58
	Procaine penicillin G	Penicillins	11	4.21	0.31	13.56
	Procaine penicillin G LA	Penicillins	3	1.68	0.25	10.87
	Sulfadoxine (with trimethoprim)	Sulfonamides	10	6.97	0.28	12.13
	Trimethoprim (with sulfadoxine)	Dihydrofolate reductase inhibitors	10	1.39	0.28	12.13
	Tulathromycin	Macrolides	1	0.12	0.40	17.51
	Tylosin	Macrolides	3	3.12	0.57	24.71

*ROA* route of administration

^a^Doses_CA_ per pig = number of Canadian defined daily doses per pig; Dose_CA_ rate = number of Canadian defined daily doses per 1000 pig-days

A greater variety of AAIs were used by injection including the Category I antimicrobials ceftiofur and enrofloxacin, than in creep feed (Table 10). Ceftiofur was given to all piglets in 4/25 herds to prevent respiratory disease, lameness, or post-processing infections. In 1/25 herds, ceftiofur was given to 5% of the piglets to prevent respiratory disease. Ceftiofur was also used for treatment of enteric disease and lameness in individual piglets in 1/25 herds. Enrofloxacin was used for treatment of clinical infections in individual piglets in 2/25 herds. The ranking of antimicrobials by quantity of use varied by metric. Procaine penicillin G was used by the highest number of herds, followed by sulfadoxine combined with trimethoprim. In mg/kg biomass the top-used AAI was sulfadoxine combined with trimethoprim, followed by procaine penicillin G and tylosin. In both defined daily dose metrics, the top AAI used was benzathine penicillin G, followed by long acting ceftiofur and tylosin. Fifty-eight percent of use by injection was for treatment purposes, against respiratory and enteric diseases, lameness, greasy pig disease, and *Streptococcus suis*. The remainder was used to prevent respiratory and enteric diseases, lameness, and post-processing infections.

#### Nursery pigs

The median length of the nursery period of 52 days (Table 2), was used as the time at risk for calculation of the animal-time denominator. The types of AAIs used in nursery feed were those used in creep feed, plus avilamycin, lincomycin and tylvalosin (Table 11). Chlortetracycline was used by the highest number of herds, followed by procaine penicillin G and tiamulin. Chlortetracycline was also the top AAI used in terms of mg/kg biomass and both dose-based metrics, followed by procaine penicillin G and sulfamethazine in mg/kg biomass, and by tylosin and sulfamethazine in defined daily dose metrics. Antimicrobial use in feed was entirely for the prevention of disease, including respiratory and enteric diseases, lameness, and *Streptococcus suis*.

**Table 11:** The quantities of antimicrobial active ingredients used in nursery feed, water, and by injection as measured in weight-based and dose-based metrics (25 nursery herds in the province of Ontario, Canada, May 2017–April 2018)

ROA	Antimicrobial active ingredients	Class	Number of herds	Mg/kg biomass	Doses_CA_/pig^a^	Dose_CA_ rate^a^
Feed	Avilamycin	Orthosomycin	1	1.2	0.4	7.2
	Chlortetracycline	Tetracyclines	18	204.3	19.7	377.9
	Lincomycin	Lincosamides	2	3.2	0.6	12.1
	Procaine penicillin G	Penicillins	8	28.9	2.2	42.1
	Sulfamethazine	Sulfonamides	6	26.0	5.9	113.5
	Tiamulin	Pleuromutilins	11	12.9	2.3	43.6
	Tylosin	Macrolides	1	20.9	6.7	129.8
	Tylvalosin	Macrolides	1	1.3	0.8	14.9
Injection	Benzathine penicillin G (with other antimicrobials)	Penicillins	2	0.01	0.007	0.14
	Enrofloxacin	Fluoroquinolones	2	0.02	0.002	0.04
	Lincomycin	Lincosamides	1	0.03	0.003	0.06
	Oxytetracycline	Tetracyclines	2	0.06	0.004	0.08
	Procaine penicillin G	Penicillins	11	1.13	0.084	1.61
	Procaine penicillin G LA	Penicillins	2	0.01	0.001	0.02
	Sulfadoxine (with trimethoprim)	Sulfonamides	2	0.43	0.018	0.34
	Trimethoprim (with sulfadoxine)	Dihydrofolate reductase inhibitors	2	0.09	0.018	0.34
	Tulathromycin	Macrolides	3	0.02	0.076	1.47
Water	Amoxicillin	Penicillins	5	32.2	2.01	38.7
	Apramycin	Aminoglycoside	2	6.9	0.69	13.3
	Penicillin G potassium	Penicillins	3	5.5	0.31	6.0
	Sulfamethazine	Sulfonamide	1	9.8	0.12	2.4
	Sulfathiazole	Sulfonamide	1	4.9	0.11	2.0
	Trimethoprim (with sulfadiazine)	Dihydrofolate reductase inhibitors	1	0.9	0.13	2.5
	Tylvalosin	Macrolides	1	1.1	0.23	4.4

*ROA* route of administration

^a^Doses_CA_ per pig = number of Canadian defined daily doses per pig; Dose_CA_ rate = number of Canadian defined daily doses per 1000 pig-days

Compared to suckling pigs, fewer AAIs were used by injection in nursery pigs (Table 11). An AAI used by injection in nursery pigs that was not used in suckling pigs was lincomycin. As in suckling piglets, enrofloxacin was used for individual pig treatments. The most frequently used AAI was procaine penicillin G followed by tulathromycin. The top AAI used by injection as measured in mg/kg biomass was procaine penicillin G, followed by trimethoprim-sulfadoxine. In defined daily doses, the top AAIs used were procaine penicillin G, followed by tulathromycin and trimethoprim-sulfadoxine. Use by injection was primarily to treat disease (96%), including respiratory, enteric, and neurologic diseases, lameness, tail biting infections, and *Streptococcus suis*.

In water, the top AAI used as measured by all metrics was amoxicillin (Table 11). After amoxicillin, penicillin G potassium and apramycin were used by the highest number of farms. The next highest quantity of use in both weight-based and dose-based metrics was apramycin followed by penicillin G potassium. Seventy-nine percent of use in water was to prevent diseases such as enteric and respiratory diseases, lameness, and *Streptococcus suis*. Twenty-one percent of use in water was to treat respiratory disease, lameness, and meningitis.

#### Grower-finisher pigs

The median length of the grower-finisher period of 112 days (Table 2), was used as the time at risk for calculation of the animal-time denominator. The types of AAIs used in grower-finisher feed were similar to nursery feed, with the addition of virginiamycin and the absence of avilamycin (Table 12). Like nursery pigs, the ranking of AAIs used in feed varied by metric (Table 12). In frequency and in mg/kg biomass the top AAI used was chlortetracycline, followed by lincomycin and tylosin. In defined daily dose metrics, the top AAI used was salinomycin, followed by lincomycin and tylosin. Seventy-seven percent of use in feed was to prevent enteric and respiratory diseases and lameness, with the remainder for growth promotion. AAIs used for growth promotion in feed included virginiamycin (Health Canada category II—high importance) and salinomycin (Health Canada category IV—low importance).

**Table 12:** The quantities of antimicrobial active ingredients used in grower-finisher feed, water, and by injection as measured in weight-based and dose-based metrics (23 grower-finisher herds in the province of Ontario, Canada, May 2017–April 2018)

ROA	Antimicrobial active ingredients	Class	Number of herds	Mg/kg biomass	Doses_CA_/pig^a^	Dose_CA_ rate^a^
Feed	Chlortetracycline	Tetracyclines	5	81.0	7.8	69.5
	Lincomycin	Lincosamides	10	66.5	13.3	118.8
	Salinomycin	Ionophores	2	13.8	13.8	122.9
	Tiamulin	Pleuromutilins	1	3.3	0.6	5.2
	Tylosin	Macrolides	3	33.6	10.8	96.71
	Tylvalosin	Macrolides	1	5.6	3.3	29.4
	Virginiamycin	Streptogramins	1	16.5	5.0	44.6
Injection	Benzathine penicillin G (with other antimicrobials)	Penicillins	1	0.01	0.009	0.076
	Florfenicol	Phenicols	1	0.05	0.007	0.062
	Lincomycin	Lincosamides	2	0.16	0.016	0.142
	Oxytetracycline	Tetracyclines	1	0.01	0.001	0.009
	Procaine penicillin G	Penicillins	4	0.02	0.017	0.150
	Procaine penicillin G LA	Penicillins	1	0.11	0.002	0.014
	Tiamulin	Pleuromutilins	1	0.02	0.002	0.017
	Tylosin	Macrolides	1	0.11	0.020	0.176
Water	Amoxicillin	Penicillins	1	4.3	0.27	2.4
	Penicillin G potassium	Penicillins	2	17.2	0.96	8.6
	Tetracycline	Tetracycline	1	3.7	0.42	3.8

*ROA* route of administration

^a^Doses_CA_ per pig = number of Canadian defined daily doses per pig; Dose_CA_ rate = number of Canadian defined daily doses per 1000 pig-days

By injection, the most frequently used AAI was procaine penicillin G, followed by lincomycin (Table 12). In mg/kg biomass the top AAI used was lincomycin, followed by procaine penicillin G and tylosin. In defined daily dose metrics, the top AAI used was tylosin, followed by procaine penicillin G and lincomycin. Eight percent of use by injection was to prevent lameness, with the remainder to treat respiratory and enteric diseases, lameness, *Streptococcus suis*, *Haemophilus parasuis*, and “poor doing”.

In water, the top AAI was penicillin G potassium as measured by all metrics, followed by amoxicillin in mg/kg biomass and tetracycline in defined daily dose metrics (Table 12). Fifty percent of use in water was for disease prevention, 50% for disease treatment. Diseases targeted for prevention included respiratory disease and *Actinobacillus suis*. Disease treatment included enteric and respiratory diseases and lameness.

## Discussion

This is the first study in Canada to describe AMU in lactating sows, suckling, and nursery pigs using both frequency and quantitative measures (weight-based and dose-based). Data on AMU have been reported annually for grower-finisher pigs by CIPARS using frequency metrics and weight-based metrics since 2006, and more recently using dose-based metrics [29]. Four previous studies conducted in Canada (in the provinces of Ontario in 1991 and 1992, Alberta in 2000, and Alberta and Saskatchewan in 2004) have described AMU in nursery and grower-finisher pigs, and in some cases sows, using a variety of frequency-based metrics including the number or percentage of farms metric used in this study [5, 7, 30, 31]. An Ontario and British Columbia study in 1999–2000 collected qualitative information on antimicrobial use in piglets, weaners, finishers and sows [6]. A previous U.S. study conducted in 2006–7 reported quantitative estimates of antimicrobial use in feed in nursery and grower-finisher pigs using the weight-based metric, total kilograms [32]. Quantitative studies of AMU in pigs are more common outside of North America [33–38].

As demonstrated in this study and others, the choice of AMU metric can have an impact on the results and interpretation of AMU analyses [39, 40]. We found the top antimicrobials used in each stage of pig production often varied among frequency, weight-based, and dose-based metrics. For example, in grower-finisher feed, lincomycin was used by the highest percentage of farms, chlortetracycline was the top antimicrobial used in mg/kg biomass, and salinomycin was the top antimicrobial used in both doses per pig, and the dose_CA_ rate_._ Differences between frequency metrics and quantitative metrics such as mg/kg biomass and defined daily doses are not unexpected, as the inputs for these calculations are quite different. Differences between the weight-based and dose-based metrics may be due to differences in the type of antimicrobial used, with some antimicrobials requiring higher doses to be effective. Switching the type of antimicrobial used from a higher dose product to a lower dose product may give the impression that use has decreased when measured in mg/kg animal biomass, when the number of doses given may have remained stable or increased. This is an important consideration when decisions are made about which metric to use when benchmarking or when antimicrobial use targets are considered. Since category I or II antimicrobials usually have lower doses, using a dose-based metric for benchmarking can help prevent users from switching from category III or IV antimicrobials to category I or II antimicrobials to try to remain below the benchmark by using a lower-dose drug.

There was producer-reported use of antimicrobials for growth promotion purposes in suckling and grower-finisher pig feed in this study. AAIs used for growth promotion in these pigs included a streptogramin and a tetracycline, considered of high and medium importance in human medicine, respectively, and an ionophore, considered of low importance to human medicine by Health Canada [12]. It is notable that during the nursery period, where rapid growth is occurring, there was no producer-reported use of antimicrobials for growth promotion, however, disease prevention after weaning may be a larger focus at this stage. Data collection for this study took place prior to December 1, 2018, when Canadian regulations came into effect prohibiting growth promotion claims on antimicrobial drug labels for medically important antimicrobials [9]. Future studies or surveillance are required to determine if this regulatory change has been followed by (1) reduction in the use of medically important antimicrobials in creep feed and grower-finisher feed, (2) change in growth promotion use of ionophores and other category IV antimicrobials of low importance to human medicine and (3) reduction in the overall quantity of AMU in pigs.

### Frequency measures

In this study population, most herds administered antimicrobials by at least one route of administration, with nursery herds having the highest frequency of administration of antimicrobials by any route, and grower-finisher herds the lowest, even though only one grower-finisher herd was on an RWA program. The most common route of administration of antimicrobials, measured as percentage of herds, was in-feed for nursery and grower-finisher pigs, and by injection for sows and suckling pigs. The injectable route of administration was most frequently used for individual animals, however, some sow herds routinely administered antimicrobials by injection to entire litters for disease prevention, a practice that was also reported in an Ontario study conducted in 1992 [7]. For nursery and grower-finisher pigs, in-water was the least frequent means of administration, however, the quantity of antimicrobial used in-water was higher than by injection due to administration to groups of pigs rather than individuals.

In suckling pigs, all creep feed that was offered was medicated with antimicrobials for the purpose of disease prevention or growth promotion. A major reason to offer creep feed is to introduce piglets to solid food prior to weaning in the hopes of making the transition to solid food easier at weaning [41]. However, studies have shown that ingestion of creep feed by piglets is highly variable, with some piglets consuming no creep feed prior to weaning [22, 41], which suggests that when creep feed is medicated with antimicrobials, some piglets may receive the intended dose, while others may receive a lower dose than intended, or no dose at all.

The percentage of farms using antimicrobials in nursery feed (76%) was lower than the earlier studies in the Canadian provinces of Ontario, Alberta, Saskatchewan [5, 30, 31] and the U.S. [42]. The percentage of farms in this study using antimicrobials in grower-finisher feed (65%) was higher than in the 1991 Ontario study in finisher pigs (grower feed not reported) [5], and lower than the 2000 Alberta [31], 2004 Alberta/Saskatchewan [30] and the 2006–7 U.S. studies [42]. The relatively lower use of antimicrobials in-water in this study was also observed in these Canadian and U.S. studies [5, 30, 31, 42]. In the Ontario/British Columbia study, none of the sampled farrow-to-finish farms used antimicrobials in water [6].

The AAI used by the most farms varied by production stage for in-feed and in-water routes of administration, but procaine penicillin was the AAI used by most herds across all production stages. The previous studies in the provinces of Ontario, Alberta, Saskatchewan, and British Columbia reported similar findings in grower-finisher (feeder) pigs [5, 30, 31], nursery pigs (weaners) [30, 31], sows [31], and across all stages (farrow-to-finish) [6], although these studies did not always distinguish between different forms of penicillin (e.g., procaine penicillin vs benzathine penicillin G). In water, our results were similar to two previous studies for grower-finisher pigs, but not for nursery pigs, where amoxicillin was used in our study more frequently than penicillin [30, 31]. The 1991 Ontario study reported tetracycline and dimetridazole (now banned for use in food-producing animals in Canada) as the most frequently used AAIs given to grower-finisher pigs in water, in contrast to penicillin G potassium in our study [5]. In feed, we found lincomycin (grower-finishers) and chlortetracycline (sows, nursery and suckling pigs) to be the most frequently used antimicrobials. The 2004 Alberta/Saskatchewan study reported similar results in feed for nursery pigs, sows, and grower-finishers [31], while the 2000 Alberta study reported similar results for nursery and suckling pigs, but different results for sows (oxytetracycline) and grower-finishers (tylosin) [30]. The 1991 Ontario study reported dimetridazole and tetracycline to be the most frequently used AAIs in grower-finisher feed [5]. Other in-feed AAIs reported by the previous studies that are now banned in Canada for use in food-producing animals include carbadox [5, 6, 31] and furazolidone [5].

### Weight and dose-based metrics

Making direct numerical comparisons of quantities of antimicrobials used with other studies can be challenging, due to differences in quantitative metrics used and/or the inputs used to calculate the metrics. Standard pig weights and standard drug doses may differ. For example, the standard pig weights used in this study differ from those used in some European studies [33, 43]. Many countries have set their own country-specific standardized drug doses, making comparisons in AMU between countries using dose-based metrics difficult [16]. Other methodologic differences make comparisons challenging such as the use of different routes of administration (e.g., grouping in-feed and in-water into one oral route of administration), different pig groupings (e.g., grouping sows and piglets together), and reporting of use by antimicrobial class instead of individual antimicrobial active ingredient [33, 36–38]. However, comparisons of overall trends and general observations may be useful.

Our initial hypothesis that higher quantities of antimicrobials would be used in the nursery phase compared to the grower-finisher phase of production was correct when AMU was measured in mg/kg biomass and the dose_CA_ rate. Similar observations were made using treatment incidence [34], a measure similar to the dose_CA_ rate, in Belgium, Germany, and France, but not in Sweden where AMU in suckling pigs was higher using this measure [33, 36, 44]. However, in our study, when AMU was measured in total kilograms and doses_CA_ per pig, the quantity of AMU was higher in grower-finisher pigs. These findings indicate that while more kilograms of antimicrobials overall were used in grower-finisher pigs and they received more doses of antimicrobial per pig, nursery pigs received more milligrams of antimicrobial per kg of pig produced and the dose rate was higher in nursery pigs, suggesting that the intensity of use is higher in the nursery phase of pig production in the province of Ontario. These findings also illustrate the value of using a variety of metrics to describe AMU in order to get a fuller picture of AMU. Antimicrobials are often used in nursery pigs after weaning, a stressful period for pigs, to prevent post-weaning infections, as observed in this study where all in-feed and the majority of in-water AMU in nursery pigs was for disease prevention. These findings suggest it may be possible to achieve substantial reductions in AMU by focusing antimicrobial stewardship plans on this stage of production, although husbandry and management practices may need to be adjusted. A 2014 study in Ireland compared the health and welfare of nursery pigs given antimicrobials in-feed for disease prevention at weaning with those that only received individual treatments of antimicrobials for clinical disease [45]. The Ireland study found that while the average daily gain and final weight of the nursery pigs was higher in the pigs receiving antimicrobials for disease prevention, no major health differences were detected between the two groups of pigs. The authors of the study concluded that, should the use of antimicrobials for disease prevention purposes be prohibited, individual treatment of pigs with clinical signs of disease could replace the administration of antimicrobials to entire groups of pigs [45]. In Europe, a recent regulation will place strict limits on the use of antimicrobials for prophylaxis (to prevent disease in healthy animals) and metaphylaxis (to control the spread of disease when some animals are infected) [46].

By injection, the quantity of AMU was highest in suckling pigs, compared to sows, nursery and grower-finisher pigs, as measured in mg/kg biomass and both defined daily dose metrics. In contrast, in total kilograms, the highest quantity of AMU by injection was in sows. Total kilograms alone, however, do not provide enough information to make useful comparisons in use [16, 47]. The metric mg/kg biomass is an improvement over total kilograms as it adjusts for differences in animal weights and numbers. A further improvement is made by adjusting for differences in drug used, and/or the length of the production cycle (time at risk), as occurs in the dose-based metrics [48]. Even though suckling pigs are small, after adjusting for weight and/or drug doses and time at risk, the quantity of use by injection as measured in mg/kg biomass and both dose-based metrics was higher in suckling pigs than sows, likely due to the routine use of injectable antimicrobials to entire litters for disease prevention, as discussed previously.

Relative to the amount of other injectable antimicrobials, the amount of category I AAIs ceftiofur (a 3^rd^ generation cephalosporin) and enrofloxacin (a fluoroquinolone), which were only used in suckling and nursery pigs, was low. The frequency of use was also low relative to other AAIs. Fluoroquinolones were approved for use in pigs in Canada in 2012, and prior to this, no Canadian studies reported the use of enrofloxacin in pigs [5–7, 30, 31]. The use of ceftiofur for individual pig treatments in piglets, nursery pigs, finishers and sows was reported in an Ontario study performed from 1999 to 2000 [6]. The 2000 Alberta study performed examined the use of antimicrobials in nursery pigs (weaners), growers, and finishers, and reported the use of ceftiofur by injection in all three pig types [31]. The 2004 Alberta/Saskatchewan study also reported the use of ceftiofur by injection in nursery pigs, grower-finishers, and sows [30]. The 1991 survey of Ontario swine producers did not report the use of cephalosporins in nursery pigs, finishers or growers [5], however, the study did not investigate the use of antimicrobials in suckling pigs. The subsequent inventory of empty medication bottles on Ontario farrow-to-finish farms in 1992, also did not identify the use of cephalosporins [7]. Third and fourth generation cephalosporin use has been reported in Belgium, France, and Germany where, similar to this study, the largest quantity of use was in suckling pigs, while smaller amounts were used in weaned and fattening pigs [36]. Similar results were also reported in the Netherlands for 3rd and 4th generation cephalosporins and fluoroquinolones, although the quantity of use of these AAIs was very small relative to the use of other AAIs [37]. The use of 3rd and 4th generation cephalosporins were not reported in Sweden [36].

While the frequency and quantity of ceftiofur use was low in this study, the use of this Health Canada category I antimicrobial to prevent disease in groups of suckling pigs is a concern, and is inconsistent with World Health Organization guidelines for the use of medically important antimicrobials in food animals [49]. The EMA categorizes 3rd generation cephalosporins in the “Restrict” category and recommends that they be considered only for treatment purposes when no other alternatives are available [13]. The use of ceftiofur for disease prevention could be compared to the disease prevention, off-label use of ceftiofur in-ovo in Canadian hatcheries which was voluntarily discontinued by hatcheries in Québec in 2005, and subsequently across Canada in 2014, over concerns about the potential relationship between high frequencies of resistance to ceftiofur in *Salmonella* Heidelberg isolates from chicken meat and ceftiofur-resistant *Salmonella* Heidelberg infections in people [50, 51]. After the use of ceftiofur *in-ovo* was discontinued in Québec, the frequency of ceftiofur resistance in *Salmonella* Heidelberg isolates from chicken and humans dropped [50]. In addition, the frequency of ceftiofur resistance in *E. coli* from chicken meat also dropped [50]. Similar results were found after the voluntary ban was extended across Canada by Chicken Farmers of Canada [51]. While ceftiofur resistance in *E. coli* isolates from pork in Canada has historically been low and recovery of *Salmonella* from pork is also low [52], there are other routes by which humans could be exposed to ceftiofur-resistant bacteria from piglets, including environmental exposure after contamination with manure from treated animals, and direct contact with pigs exposed to ceftiofur [53, 54]. Since this study was completed, the Canadian pork industry has taken steps improve the stewardship of Category I antimicrobials (including ceftiofur) on pig farms. The new Canadian Pork Excellence Program (updating and replacing the Canadian Quality Assurance Program), sets standards for food safety, animal care, and traceability on pig farms, and includes a new statement limiting the use of Category I antimicrobials to disease treatment only, and only when prescribed by a veterinarian [55, 56].

### Other observations

The high use of biosecurity practices, such as the use of barn-specific boots and coveralls, Danish entry system, restricting visitors to the farm, and a biosecurity sign at the farm entrance was encouraging. Biosecurity practices are the means by which producers attempt to prevent infections from entering and spreading in their herd. The low use of boot dips was not surprising, as they are generally ineffective in a farm environment [57]. The practice of quarantining new gilt introductions to sow herds was not used as frequently as other practices, possibly due to lack of quarantine facilities on some farms and/or the replacement of gilts with the herd’s own stock. It was beyond the scope of this study to evaluate the effectiveness of these practices, however, the use of biosecurity practices has been linked to decreased AMU, which suggests that if the use of biosecurity practices had been lower than observed in this study, the quantity of AMU may have been higher [43, 58].

Fully investigating the health and vaccination data collected in relation to the type and quantity of AMU was beyond the scope of this study. We did note that for some diseases/disease agents, the number of farrowing herds reporting a positive disease status in lactating sows matched the number of herds reporting a positive disease status in suckling pigs, including pathogenic *E. coli*, PCVAD, and *Streptococcus suis*. Disease from pathogenic *E. coli* tends to be more common in young pigs; hence, it is notable that 92% of herds were considered positive for *E. coli* in lactating sows, compared to 2% in grower-finisher pigs. In this study, we considered a herd to be positive for a particular disease/disease agent whether or not laboratory confirmation of infection was performed. In lactating sows, six of the 23 positive *E. coli* herds reported a “confirmed positive” disease status, meaning the diagnosis was confirmed with laboratory testing, while the remaining 17 herds were “likely positive”. It is possible that clinical disease due to *E. coli* is higher than expected in lactating sows, or that veterinarians and/or producers consider sows to be “likely positive” for *E. coli* when the suckling pigs are infected, even if the sows do not develop clinical disease. Over 75% of the farrowing herds were considered positive for PCVAD in both lactating sows and suckling pigs, despite vaccination of the herds against porcine circovirus type 2 in 68% of herds in lactating sows and 48% of herds in suckling piglets. Vaccination against *S. suis* may be low due to a lack of effective vaccines. Similarly, in the nursery, over 75% of the nursery herds were considered positive for pathogenic *E. coli*, PCVAD, and *S. suis*, as well as *H. parasuis*. Building on the health information collected as part of this study with more detailed investigations into the health status of pig herds for these common diseases would be useful to improve our understanding of disease pressures and to explore where opportunities to reduce the need for antimicrobials might exist. It is important to acknowledge that without the high frequency of use of most biosecurity practices reported by herds in this study, the positivity for diseases/disease agents may have been higher.

While piglets are commonly weaned in Canada at 21 days of age, some piglets in this study were transferred to the nursery at 18 days of age, while others spent up to 41 days in the farrowing unit. In some cases, we speculate that when it comes time to wean and move a new batch of piglets to the nursery, some piglets in the batch may fall below the targeted weaning age. Younger piglets likely have a more difficult transition at weaning than older piglets, which may affect antimicrobial use. In Canada, herds in organic or welfare-friendly programs are often required to wean at older ages (potentially up to 6–8 weeks of age). Organic herds were not included in this study, as we used the same inclusion and exclusion criteria as the CIPARS Farm Surveillance component, which excludes organic herds [4]. However, some herds in the study may be in the process of transitioning to an organic program or may be part of a welfare-friendly program. In a subsequent analysis, we plan to include weaning age in an investigation of factors that may affect the quantity of AMU in the herds included in this study. Further investigation into common weaning ages in piglets in Canada would be useful for a greater understanding of weaning age and antimicrobial use in newly weaned pigs.

It is possible that the findings of this study were influenced by the method of selection of farms, as participation by producers was voluntary. Producers interested in antimicrobial use and stewardship may have been more likely to participate than others. We attempted to minimize selection bias by asking the contracted veterinarians to select farms that were representative of the range of farms in their practice, however, we still relied on producers’ willingness to participate. Without selection bias and with a larger sample of farms we may have observed a greater range of biosecurity and antimicrobial use practices. Nevertheless, as this is the first study in Canada to report AMU from birth to slaughter in both quantitative and qualitative terms, its findings will be useful for the development of good antimicrobial stewardship practices and for designing future studies in this area.

## Conclusions

This study provided a current picture of which antimicrobials are in use and the quantity of AMU on a purposive sample of sows, suckling, nursery, and grower-finisher pigs on swine herds in the province of Ontario, Canada. We were encouraged by the high frequency of use of biosecurity practices among the participating farms. We noted that the highest quantity of AMU was administered in feed (compared to in water and by injection), that more antimicrobials were used at the nursery stage relative to the farrowing and grower-finisher stages (although ranking of production stages was affected by the metric used), that there was routine use of injectable antimicrobials of very high importance in human medicine, and that medically important antimicrobials were used for growth promotion in suckling and grower-finisher feed (the study was conducted prior to the December 2018 prohibition of growth promotion claims for medically important antimicrobials. There was a wide range of weaning ages and health status findings that warrant further investigation, such as herd positivity for pathogenic *E. coli* in lactating sows. Also of interest was the effect of metric choice on the ranking of antimicrobials and production stages by quantity of use. As we observed in this study, the choice of metric used to quantify AMU can influence the results obtained, which could influence subsequent antimicrobial stewardship decisions.

The findings of this study will help provide a basis for further investigation into AMU in pigs in Ontario, and in the other major pig-producing provinces of Canada (including Québec, Manitoba, and Saskatchewan), with the goal of better understanding the need for antimicrobials in pig production in Canada and exploring potential opportunities for improvements in antimicrobial stewardship, where possible. We conclude that it would be beneficial to include the farrowing and nursery stages of pig production in routine surveillance of on-farm antimicrobial use, while acknowledging that expanding existing surveillance programs to these production stages would require additional infrastructure and financial resources.

The information provided by this study will be useful for designing future studies and surveillance programs examining AMU in pigs across Canada and other jurisdictions. Now that we have current information on which antimicrobials are in use, there are opportunities to investigate more targeted questions, such as why certain antimicrobials are being used for specific purposes, and to what degree antimicrobial choices are based on laboratory diagnoses and antimicrobial susceptibility testing. We have future research planned based on the data collected in this study, including an examination of associations between the quantity of AMU and factors such as herd size, operation type (all-in-all-out versus continuous flow), enrolment in RWA programs, weaning age, health status, and other factors of interest. We also plan to examine antimicrobial and route of administration choices and reasons for use (growth promotion, disease prevention and disease treatment).

## Supplementary Information

**Additional file 1**. The farrowing questionnaire used to collect information on antimicrobial use, biosecurity practices, health status, and animal numbers in Ontario sow herds, May 2017–April 2018. An English copy of the farrowing questionnaire used to collect data from participating sow herds in Ontario, Canada (May 2017–April 2018).

**Additional file 2**. The nursery questionnaire used to collect information on antimicrobial use, biosecurity practices, health status, and animal numbers in Ontario nursery pig herds, May 2017–April 2018. An English copy of the farrowing questionnaire used to collect data from participating nursery pig herds in Ontario, Canada (May 2017–April 2018).

## Data Availability

The datasets supporting the conclusions of this article are available from the corresponding author on reasonable request. French versions of the questionnaires are also available from the corresponding author on reasonable request.
