# Supplementary material for: Antimicrobial use in lactating sows, piglets, nursery, and grower-finisher pigs on swine farms in Ontario, Canada during 2017 and 2018
Source: Porcine Health Manag. 2022 Apr 28;8:17. doi: 10.1186/s40813-022-00259-w (PMC9047262; doi:10.1186/s40813-022-00259-w)
Supplement: Supplementary file 1 — Additional file 1. The farrowing questionnaire used to collect information on antimicrobial use, biosecurity practices, health status, and animal numbers in Ontario sow herds, May 2017–April 2018. An English copy of the farrowing questionnaire used to collect data from participating sow herds in Ontario, Canada (May 2017–April 2018). [file 40813_2022_259_MOESM1_ESM.pdf]

Herd ID Code:
Vet. ID Code:

CANADIAN INTEGRATED PROGRAM FOR ANTIMICROBIAL RESISTANCE SURVEILLANCE (CIPARS)[1]

NURSING/NURSERY PIG ANTIMICROBIAL USE RESEARCH PROJECT

QUESTIONNAIRE

FARROWING OPERATIONS

INSTRUCTIONS:

PLEASE READ CAREFULLY

- ❑ If you do not know how to answer any question **please feel free to contact:**

  Louise Bellai: 519-826-2348

- ❑ Complete this questionnaire based on a single farrowing period.
- ❑ **PLEASE SAMPLE BEFORE A SUBSTANTIAL NUMBER OF PIGLETS FROM THE GROUP HAVE BEEN TRANSFERRED TO THE NURSERY**
- ❑ **Piglets sampled today should:**
  - Be within 7 days of weaning;
  - Be nursing on the sow.
- ❑ **The farrowing area must be defined in Question 2. All subsequent questions must be answered on this basis (barn or room).**
- ❑ For your reference, an **appendix** listing some of the veterinary antimicrobial products available for use in swine can be found at the end of the questionnaire.
- ❑ Enter the **Herd ID** and **Vet ID** codes in the boxes on the top right corner of **each page**.
- ❑ **Please answer all questions**
  - If the producer does not know the answer, indicate "D/K" as the response;
  - If a question is not applicable to the herd, indicate "N/A" as the response.
- ❑ This questionnaire is in triplicate
  - **White** copy: Send to CIPARS in the express post envelope provided;
  - **Yellow** copy: Retained by the herd veterinarian;
  - **Pink** copy: Retained by the owner of the barn.

---

[1] CIPARS – A national program that monitors antimicrobial use and resistance.

**Herd ID Code:**
**Vet. ID Code:**

REPORTING INFORMATION

1. **Date** this questionnaire was completed and samples collected: ______ / ______ / ______
Month Day Year

2. **Farrowing Area***: All data, including piglet inventory for this questionnaire are provided by the: ❑ Room ❑ Barn
(*Check only one please*)

***Questions 9 through 20 must all refer to the same farrowing group in the farrowing area.**

For this questionnaire, **farrowing group** refers to piglets born to sows that have moved into the farrowing area as a group, and have farrowed within a period of approximately seven days.

3. **Dates and samples that these data pertain to:**

Continuous flow operations: (*The entire room/barn **is not** emptied of sows and nursing piglets at the end of each farrowing period; at least one or more pig(s) remain in the room/barn*.). The start date would be today's date minus the average weaning age, as indicated in question 9A.

All-in-all-out operations: (*The entire room/barn **is** emptied of sows and nursing piglets at the end of each farrowing period, **no pigs** remain in the room/barn*.) The start date would be the day that the piglets sampled today were born. The projected end date is the date when it is anticipated that all of the piglets from the same farrowing group as the piglets sampled today will be weaned, as indicated in question 9A.

A. Start date: ______ / ______ / ______
Month Day Year

B. End date for **Continuous Flow** operations is today's date: ______ / ______ / ______
Month Day Year

C. Projected end date for **All-In-All-Out** operations: ______ / ______ / ______
Month Day Year

White copy: Public Health Agency; **Yellow copy**: Herd Veterinarian; **Pink copy**: Producer

SAMPLE INFORMATION

D. Please complete the table below for the six farrowing crates/pens sampled today:

***REMINDER**

Piglets sampled today should:

- Be within 7 days of weaning;
- Be nursing on the sow.
- The six (6) farrowing crates/pens sampled today should be located in the same farrowing area (as defined in Question 2).

**Sample ID Code**	**Piglet Age** (in days)
	Days
	Days
	Days
	Days
	Days
	Days

Herd ID Code:

Vet. ID Code:

GENERAL HERD AND SITE INFORMATION

4. Herd Information:

A. Is the farrowing operation sampled today:
❍ Independent ❍ Part of a production group ❍ Don't Know

B. Do you have a nursery operation?
❍ Yes ❍ No

If **yes**:

i. Is it: ❍ On-site ❍ Off-Site

ii. Is it participating in this project: ❍ Yes ❍ No

If yes, please provide the nursery Herd ID ____________________

C. Do you have a grower-finisher operation?
❍ Yes ❍ No

If ***yes***:

i. Is it: ❍ On-site ❍ Off-Site

ii. Is it a CIPARS herd: ❍ Yes ❍ No

If yes, please provide the grower-finisher Herd ID ____________________

5. What is the total number of sows at this site?

____________ Sows

**Herd ID Code:**	
**Vet. Id Code:**	

6. Are the pigs sampled today part of a "Raised without Antibiotics" (RWA) production system? ❍ Yes ❍ No ❍ Don't Know

A. If RWA, what was the last date when antibiotics were used in nursing piglets in this system? ______ / ______ / ______
Month Day Year

B. If RWA, are antibiotic treatments permitted in boars and gestating sows? ❍ Yes ❍ No ❍ Don't Know

C. If RWA, are antibiotic treatments permitted in lactating sows? ❍ Yes ❍ No ❍ Don't Know

7. **Level of Biosecurity:**

Boots provided by farm:	❍Yes	❍No		
Coveralls provided by farm:	❍Yes	❍No		
Boot dip:	❍Yes	❍No		
Biosecurity Sign:	❍Yes	❍No		
Danish Entry	❍Yes	❍No		
Locked Doors	❍Yes	❍No		
Restricting visitors	❍Yes	❍No		
Shower:	❍Yes	❍No		
Quarantine of new gilts:	❍Yes	❍No		
Downtime*:	❍Yes	❍No	Hours of downtime:	
Other:	❍Yes	❍No	Specify:	

***Note:** Downtime here refers to the requirement for visitors and personnel to refrain from visiting the farm for a certain length of time after contact with other pigs/swine farms.

8. **Number of pig farms located within two kilometres of this site:** ________ Farm(s)

NURSING PIGLET INFORMATION

ATTENTION:

- All questionnaire data, including pig numbers must correspond to the same farrowing group and farrowing area, as defined in question 2.
- **These estimates are critical to our analysis.**

---

9. Nursing Piglet Numbers

CONTINUOUS FLOW OPERATIONS (*For All-In-All-Out operations please proceed to the following page*)

A. Farrowing period: What is the average number of days that piglets are in the farrowing phase of production? (What is the average age, in days, at weaning?)

____________ Days

The following questions (9B through 9E) refer to the timeframe defined by today's date minus the average age at weaning, as indicated above in 9A.

B. Piglets born alive: Estimate the total number of piglets that were born alive during the total number of days specified above in 9A. ____________ Piglets

C. Pre-weaning Mortality: Estimate the number OR percent of mortalities in this farrowing area (Q.2) during the number of days specified above in 9A

____________ Percent

OR

____________ Piglets

D. Pigs Weaned: Estimate the number of piglets weaned from this farrowing area, during the number of days specified above in 9A. ____________ Piglets

E. Pigs today: Estimate the number of piglets that are in this farrowing area today. ____________ Piglets

F. What is the number of farrowing crates/pens in this farrowing area? ____________ Crates/pens

9. Nursing Piglet Numbers

ALL-IN-ALL-OUT OPERATIONS

A. **Farrowing period:** What is the average number of days that the piglets sampled today will spend in the farrowing phase of production (What is the average age in days at weaning)? ___________ Days

B. **Piglets born alive**: Estimate the number of piglets that were born alive in this farrowing area (see Q. 2). ___________ Piglets

C. **Pre-weaning Mortality**: Estimate the number or percent of mortalities in this farrowing area (Q.2). ___________ Percent

OR

___________ Piglets

D. **Piglets today***: Estimate the number of piglets in this farrowing area today. ___________ Piglets*

*__Note:__ The answer for 9D should equal the # of pigs in 9B minus the mortality in 9C.

E. What is the number of farrowing crates/pens in this farrowing barn? ___________ Crates/pens

Herd ID Code:
Vet. ID Code:

Attention:

- **The following applies to the feed, water, oral, and injectable antimicrobial information tables for both the nursing piglets and the lactating sows:**

For both **Continuous Flow** and **All-In-All-Out** operations these data pertain to the farrowing group that was specified in Questions 3 and 9, and the farrowing area specified in Question 2.

Disease Treatment: an antimicrobial was started because a pig in the barn was suffering from a disease or condition of concern.

Disease Prevention: an antimicrobial was started but no pig was sick at that time.

Growth Promotion: an antimicrobial was used to improve growth or feed efficiency only.

Pulsed medication: a repetitive short-term medication protocol, e.g. 3 days of medication followed by 7 days without medication, repeated 3 times

10. Creep Feed Information

A. Do you offer creep feed? ❍Yes ❍No

IF NO, GO TO QUESTION 11. IF YES, PLEASE CONTINUE BELOW.

B. At what age (in days) do you start offering creep feed? ________________ Days

C. What percentage of creep feed do you estimate is wasted? ________________ Percent

Herd ID Code:

Vet. ID Code:

10. Creep feed information continued

D. Medicated Creep Feed Use in Nursing Piglets.

Check here ❍ if no antimicrobials were given in creep feed to nursing piglets during this period.

**Age of Piglets at Start** (days)	**Age of Piglets at End** (days)	**Primary Reason For Medication Use** *Choose **only one** primary reason by checking "Yes": Growth promotion OR Disease prevention OR Treatment*			**Name of Active Antimicrobial Ingredient(s)** **See the Appendix for assistance*	**Grams of Active Ingredient per Tonne** (g/tonne)	**Percent of Piglets Fed**: Estimate the % of piglets that were medicated in this farrowing area.
		**Growth Promotion**	**Disease Prevention** (If Yes, *check all disease checkboxes that apply)*	**Disease Treatment** (If Yes, *check all disease checkboxes that apply)*			
days	days	❍Yes	❍Yes: ❑ Respiratory disease ❑ Enteric disease ❑ Lameness ❑ Other (Specify)	❍Yes: ❑ Respiratory disease ❑ Enteric disease ❑ Lameness ❑ Other (Specify)		g/tonne	
days	days	❍Yes	❍Yes: ❑ Respiratory disease ❑ Enteric disease ❑ Lameness ❑ Other (Specify)	❍Yes: ❑ Respiratory disease ❑ Enteric disease ❑ Lameness ❑ Other (Specify)		g/tonne	
days	days	❍Yes	❍Yes: ❑ Respiratory disease ❑ Enteric disease ❑ Lameness ❑ Other (Specify)	❍Yes: ❑ Respiratory disease ❑ Enteric disease ❑ Lameness ❑ Other (Specify)		g/tonne	
days	days	❍Yes	❍Yes: ❑ Respiratory disease ❑ Enteric disease ❑ Lameness ❑ Other (Specify)	❍Yes: ❑ Respiratory disease ❑ Enteric disease ❑ Lameness ❑ Other (Specify)		g/tonne	

**FARM SWINE QUESTIONNAIRE**
CIPARS.FS.2017<br>**FARROWING PERIOD**

**Herd ID Code:**
**Vet. ID Code:**

11. Individual Oral Antibiotic Use in Nursing Piglets

Check here ❍ if no individual oral antibiotics were given to nursing piglets during this period.

IMPORTANT: If for one of the active ingredients listed there is more than one type of use, then fill in a **new line** for each type of use.
For example if the same antimicrobial(s) are given:

- **At different ages (e.g. at processing and again later in the farrowing period)**
- **For different disease indications**

**Product Name and Concentration** (mg/pkg, mg/g, mg/ml, mg/pump, other)<br>**Please indicate units*	**Name of Active Antimicrobial Ingredient(s)**<br>**See the Appendix for assistance*	**Volume Given to Each Piglet per Day** (mls, pump or other)<br>*Please indicate units	**Number of Days Given**	**Average Age at Start of Treatment** (days)	**Average Weight at Start of Treatment** (kgs)	**Primary Reason For Medication Use**<br>Choose **only one** primary reason<br>*Disease prevention OR Disease treatment*<br>**If Disease Prevention** (*Check all that apply*)	 <br><br><br>**If Disease Treatment** (*Check all that apply*)	**Percent of Piglets Exposed**: Estimate the % of nursing piglets in this farrowing area that were medicated for the number of days indicated.
**Name:**<br>**Concentration:** ❍ mg/ml ❍ mg/g ❍ mg/pkg ❍ mg/pump ❍ Other (Specify): ________			days	days	kgs	❑ Respiratory disease<br>❑ Enteric disease<br>❑ Lameness<br>❑ Other (Specify):	❑ Respiratory disease<br>❑ Enteric disease<br>❑ Lameness<br>❑ Other (Specify):	
**Name:**<br>**Concentration:** ❍ mg/ml ❍ mg/g ❍ mg/pkg ❍ mg/pump ❍ Other (Specify): ________			days	days	kgs	❑ Respiratory disease<br>❑ Enteric disease<br>❑ Lameness<br>❑ Other (Specify):	❑ Respiratory disease<br>❑ Enteric disease<br>❑ Lameness<br>❑ Other (Specify):	

This *Individual Oral Antibiotic in Nursing Piglets table* is continued on the following page.

FARM SWINE QUESTIONNAIRE

CIPARS.FS.2017
FARROWING PERIOD

Herd ID Code:

Vet. ID Code:

Individual Oral Antibiotic in Nursing Piglets table continued.

**Product Name and Concentration** (mg/pkg, mg/g, mg/ml, mg/pump, other)<br>**Please indicate units*	**Name of Active Antimicrobial Ingredient(s)**<br>**See the Appendix for assistance*	**Volume Given to Each Piglet per Day** (mls, pump or other)<br>*Please indicate units	**Number of Days Given**	**Average Age at Start of Treatment** (days)	**Average Weight at Start of Treatment** (kgs)	**Primary Reason For Medication Use** Choose **only one** primary reason *Disease prevention OR Disease treatment*<br>**If Disease Prevention** (*Check all that apply)*	 **If Disease Treatment** *(Check all that apply)*	**Percent of Piglets Exposed**: Estimate the % of nursing piglets in this farrowing area that were medicated for the number of days indicated.
**Name:**<br><br>**Concentration:** ❍ mg/ml ❍ mg/g ❍ mg/pkg ❍ mg/pump ❍ Other (Specify): ________<br>__________			days	days	kgs	❑ Respiratory disease<br>❑ Enteric disease<br>❑ Lameness<br>❑ Other (Specify):	❑ Respiratory disease<br>❑ Enteric disease<br>❑ Lameness<br>❑ Other (Specify):	
**Name:**<br><br>**Concentration:** ❍ mg/ml ❍ mg/g ❍ mg/pkg ❍ mg/pump ❍ Other (Specify): ________<br>__________			days	days	kgs	❑ Respiratory disease<br>❑ Enteric disease<br>❑ Lameness<br>❑ Other (Specify):	❑ Respiratory disease<br>❑ Enteric disease<br>❑ Lameness<br>❑ Other (Specify):	
**Name:**<br><br>**Concentration:** ❍ mg/ml ❍ mg/g ❍ mg/pkg ❍ mg/pump ❍ Other (Specify): ________<br>__________			days	days	kgs	❑ Respiratory disease<br>❑ Enteric disease<br>❑ Lameness<br>❑ Other (Specify):	❑ Respiratory disease<br>❑ Enteric disease<br>❑ Lameness<br>❑ Other (Specify):	

FARM SWINE QUESTIONNAIRE	Herd ID Code:
CIPARS.FS.2017<br>**FARROWING PERIOD**	**Vet. ID Code:**

12. Injectable Antibiotic Use in Nursing Piglets. List continued on the following pages.

Check here ❍ if no injectable antibiotics were given to nursing piglets during this period.

IMPORTANT: If for one of the active ingredients listed there is more than one type of use, then fill in a **new line** for each type of use.
For example if the same antimicrobial(s) are given:

- **At different ages (e.g. at processing and again later in the farrowing period)**
- **For different disease indications**

**Product Name and Concentration** (mg/ml)	**Name of Active Antimicrobial Ingredient(s)** **See the Appendix for assistance*	**Volume Given to Each Piglet per Day** (mls)	**Number of Days Given**	**Average Age at Start of Treatment** (days)	**Average Weight at Start of Treatment** (kgs)	**Primary Reason For Medication Use** Choose **only one** primary reason *Disease prevention OR Disease treatment*		**Percent of Piglets Exposed**: Estimate the % of nursing piglets in this farrowing area that were medicated for the number of days indicated.
						**If Disease Prevention** *(Check all that apply)*	**If Disease Treatment** *(Check all that apply)*	
**Name:**<br><br>**mg/ml:**		mls	days	days	kgs	❑ Respiratory disease<br>❑ Enteric disease<br>❑ Lameness<br>❑ Other (Specify):	❑ Respiratory disease<br>❑ Enteric disease<br>❑ Lameness<br>❑ Other (Specify):	
**Name:**<br><br>**mg/ml:**		mls	days	days	kgs	❑ Respiratory disease<br>❑ Enteric disease<br>❑ Lameness<br>❑ Other (Specify):	❑ Respiratory disease<br>❑ Enteric disease<br>❑ Lameness<br>❑ Other (Specify):	
**Name:**<br><br>**mg/ml:**		mls	days	days	kgs	❑ Respiratory disease<br>❑ Enteric disease<br>❑ Lameness<br>❑ Other (Specify):	❑ Respiratory disease<br>❑ Enteric disease<br>❑ Lameness<br>❑ Other (Specify):	

This *Injectable Antibiotics in Nursing Piglets table* is continued on the following page.

FARM SWINE QUESTIONNAIRE

CIPARS.FS.2017
FARROWING PERIOD

Herd ID Code:

Vet. ID Code:

Injectable Antibiotics in Nursing Piglets table continued.

**Product Name and Concentration** (mg/ml)	**Name of Active Antimicrobial Ingredient(s)** **See the Appendix for assistance*	**Volume Given to Each Piglet per Day** (mls)	**Number of Days Given**	**Average Age at Start of Treatment** (days)	**Average Weight at Start of Treatment** (kgs)	**Primary Reason For Medication Use** Choose **only one** primary reason *Disease prevention OR Disease treatment*		**Percent of Piglets Exposed**: Estimate the % of nursing piglets in this farrowing area that were medicated for the number of days indicated.
						**If Disease Prevention** (*Check all that apply*)	**If Disease Treatment** (*Check all that apply*)	
**Name:** **mg/ml:**		mls	days	days	kgs	❑ Respiratory disease ❑ Enteric disease ❑ Lameness ❑ Other (Specify):	❑ Respiratory disease ❑ Enteric disease ❑ Lameness ❑ Other (Specify):	
**Name:** **mg/ml:**		mls	days	days	kgs	❑ Respiratory disease ❑ Enteric disease ❑ Lameness ❑ Other (Specify):	❑ Respiratory disease ❑ Enteric disease ❑ Lameness ❑ Other (Specify):	
**Name:** **mg/ml:**		mls	days	days	kgs	❑ Respiratory disease ❑ Enteric disease ❑ Lameness ❑ Other (Specify):	❑ Respiratory disease ❑ Enteric disease ❑ Lameness ❑ Other (Specify):	
**Name:** **mg/ml:**		mls	days	days	kgs	❑ Respiratory disease ❑ Enteric disease ❑ Lameness ❑ Other (Specify):	❑ Respiratory disease ❑ Enteric disease ❑ Lameness ❑ Other (Specify):	

Herd ID Code:

Vet. ID Code:

SOW INFORMATION

13. **Sow Numbers:** How many sows are in this farrowing area (Q2) with the piglets sampled today? ___________ Sows

14. **Sow Parity:**

A. What is the minimum and maximum parity of the sows in this farrowing area? ___________ Minimum ___________ Maximum

B. What is the estimated average parity of the sows in this farrowing area? ___________ Parity

15. **LIST ALL THE RATIONS used to feed sows in this farrowing area during the farrowing period that was specified in questions 3 and 9.**

A farrowing period is the total number of weeks that piglets and sows are in the farrowing unit. In most barns this would be a 3 to 4 week period.

Attention: These data are critical to our analysis

Ration Name	Minimum Sow Weight	Maximum Sow Weight	Average # Weeks Fed per Farrowing Period
	Circle: Kgs or Lbs		
**NOTE:** THIS TOTAL SHOULD EQUAL THE NUMBER OF WEEKS THAT IT TAKES THE AVERAGE SOW TO GO FROM THE START OF THE FARROWING PERIOD TO THE END (WEANING). ⇨		**Total # of weeks that sows are in the farrowing unit:**	

Herd ID Code:

Vet. ID Code:

16. Medicated Feed Use in Sows in the Farrowing Area

IN THE TABLE BELOW COMPLETE ONE LINE FOR EACH RATION FED TO SOWS IN THE FARROWING AREA INCLUDING NON-MEDICATED RATIONS.
NOTE: The ration names provided in the table above (Question 15**) MUST correspond** to the ration names used in the table below**.**

IMPORTANT: Sow feed information provided below should be for the same farrowing area indicated in Question 2 (Reporting level: Barn or Room) and for the farrowing period indicated in Question 3.

If for one of the rations listed in Question 15 there is ANY change in MEDICATION then fill in a new line for each change e.g. medicated to non-medicated, change in inclusion rates, change in drug incorporated. You do not need to start a new line if there is a change in ration formulation from a nutrient perspective. A new line is only needed for medication changes. **Additional space** is available over the **next page**.

Attention: These data are critical to our analysis. Please fill in all of the information requested.

**Ration Name** (from Question 15)	**Medicated?**	**Primary Reason For Medication Use** *Choose **only one** primary reason by checking "Yes": Disease prevention OR Treatment*		**Name of Active Antimicrobial Ingredient(s)** **See the Appendix for assistance*	**Grams of Active Ingredient per Tonne** (g/tonne)	**Percent of Sows Fed**: Estimate the % of sows in this farrowing area that were fed each ration for number of weeks indicated in Q15.
		**Disease Prevention** (If Yes, *check all disease checkboxes that apply)*	**Disease Treatment** (If Yes, *check all disease checkboxes that apply)*			
	❍ Yes<br>❍ No<br>❑ Pulsed<br>Total Days: ______	❍Yes:<br>❑ Respiratory disease<br>❑ Enteric disease<br>❑ Lameness<br>❑ Other (Specify):	❍Yes:<br>❑ Respiratory disease<br>❑ Enteric disease<br>❑ Lameness<br>❑ Other (Specify):		(g/tonne)	
	❍ Yes<br>❍ No<br>❑ Pulsed<br>Total Days: ______	❍Yes:<br>❑ Respiratory disease<br>❑ Enteric disease<br>❑ Lameness<br>❑ Other (Specify):	❍Yes:<br>❑ Respiratory disease<br>❑ Enteric disease<br>❑ Lameness<br>❑ Other (Specify):		(g/tonne)	

This *Sow Medicated Feed table* is continued on the following page.

Medicated feed in sows table continued.

**Ration Name** (from Question 15)	**Medicated?**	**<u>Primary</u> Reason For Medication Use** *Choose **only one** primary reason by checking "Yes": Disease prevention OR Treatment*		**Name of Active Antimicrobial Ingredient(s)** **See the Appendix for assistance*	**Grams of Active Ingredient per Tonne** (g/tonne)	**Percent of Sows Fed**: Estimate the % of sows in this farrowing area that were fed each ration for number of weeks indicated in Q.15.
		**Disease Prevention** (If Yes, *check all disease checkboxes that apply)*	**Disease Treatment** (If Yes, *check all disease checkboxes that apply)*			
	❍ Yes<br>❍ No<br>❑ Pulsed<br>Total Days: ______	❍Yes:<br>❑ Respiratory disease<br>❑ Enteric disease<br>❑ Lameness<br>❑ Other (Specify):	❍Yes:<br>❑ Respiratory disease<br>❑ Enteric disease<br>❑ Lameness<br>❑ Other (Specify):		(g/tonne)	
	❍ Yes<br>❍ No<br>❑ Pulsed<br>Total Days: ______	❍Yes:<br>❑ Respiratory disease<br>❑ Enteric disease<br>❑ Lameness<br>❑ Other (Specify):	❍Yes:<br>❑ Respiratory disease<br>❑ Enteric disease<br>❑ Lameness<br>❑ Other (Specify):		(g/tonne)	
	❍ Yes<br>❍ No<br>❑ Pulsed<br>Total Days: ______	❍Yes:<br>❑ Respiratory disease<br>❑ Enteric disease<br>❑ Lameness<br>❑ Other (Specify):	❍Yes:<br>❑ Respiratory disease<br>❑ Enteric disease<br>❑ Lameness<br>❑ Other (Specify):		(g/tonne)	
	❍ Yes<br>❍ No<br>❑ Pulsed<br>Total Days: ______	❍Yes:<br>❑ Respiratory disease<br>❑ Enteric disease<br>❑ Lameness<br>❑ Other (Specify):	❍Yes:<br>❑ Respiratory disease<br>❑ Enteric disease<br>❑ Lameness<br>❑ Other (Specify):		(g/tonne)	

17. Medicated Water Use in Sows in the Farrowing Area

Check here ❍ if no antibiotics were administered in water to sows in this farrowing area during this farrowing period.

IMPORTANT: If for one of the active ingredients listed there is more than one type of use, then fill in a **new line** for each type of use.
For example if the same antimicrobial(s) are given:

- **At different ages (e.g. at farrowing and again later in the farrowing period)**
- **For different disease indications**

**Product Name and Concentration** (mg/pkg, mg/g, IU/g, other) *Please indicate units	**Name of Active Antimicrobial Ingredient(s)** *See the Appendix for assistance	**Grams of Active Ingredient per Litre of Water** (g/L)	**Number of Days Given**	**Primary Reason For Medication Use** Choose **only one** primary reason *Disease prevention OR Disease treatment*		**Percent of Sows Exposed**: Estimate the % of sows in this farrowing area that were medicated for the number of days indicated.
				**If Disease Prevention** (*Check all that apply*)	**If Disease Treatment** (*Check all that apply*)	
**Name:** ______<br>**Concentration:** ❍ mg/g ❍ mg/pkg ❍ IU/g ❍ Other (Specify): ______		g/L	days	❑ Respiratory disease<br>❑ Enteric disease<br>❑ Lameness<br>❑ Other (Specify):	❑ Respiratory disease<br>❑ Enteric disease<br>❑ Lameness<br>❑ Other (Specify):	
**Name:** ______<br>**Concentration:** ❍ mg/g ❍ mg/pkg ❍ IU/g ❍ Other (Specify): ______		g/L	days	❑ Respiratory disease<br>❑ Enteric disease<br>❑ Lameness<br>❑ Other (Specify):	❑ Respiratory disease<br>❑ Enteric disease<br>❑ Lameness<br>❑ Other (Specify):	

This *Medicated Water in Sows table* is continued on the following page.

FARM SWINE QUESTIONNAIRE

CIPARS.FS.2017
FARROWING PERIOD

Herd ID Code:

Vet. ID Code:

Medicated water in Sows table continued.

**Product Name and Concentration** (mg/pkg, mg/g, IU/g, other)<br>*Please indicate units*	**Name of Active Antimicrobial Ingredient(s)**<br>*See the Appendix for assistance*	**Grams of Active Ingredient per Litre of Water** (g/L)	**Number of Days Given**	**Primary Reason For Medication Use** Choose **only one** primary reason *Disease prevention OR Disease treatment*<br>**If Disease Prevention** (Check all that apply)	**If Disease Treatment** (Check all that apply)	**Percent of Sows Exposed**: Estimate the % of sows in this farrowing area that were medicated for the number of days indicated.
**Name:**<br>**Concentration:** ❍ mg/g ❍ mg/pkg ❍ IU/g ❍ Other (Specify):		g/L	days	❑ Respiratory disease<br>❑ Enteric disease<br>❑ Lameness<br>❑ Other (Specify):	❑ Respiratory disease<br>❑ Enteric disease<br>❑ Lameness<br>❑ Other (Specify):	
**Name:**<br>**Concentration:** ❍ mg/g ❍ mg/pkg ❍ IU/g ❍ Other (Specify):		g/L	days	❑ Respiratory disease<br>❑ Enteric disease<br>❑ Lameness<br>❑ Other (Specify):	❑ Respiratory disease<br>❑ Enteric disease<br>❑ Lameness<br>❑ Other (Specify):	
**Name:**<br>**Concentration:** ❍ mg/g ❍ mg/pkg ❍ IU/g ❍ Other (Specify):		g/L	days	❑ Respiratory disease<br>❑ Enteric disease<br>❑ Lameness<br>❑ Other (Specify):	❑ Respiratory disease<br>❑ Enteric disease<br>❑ Lameness<br>❑ Other (Specify):	

Herd ID Code:

Vet. ID Code:

18. INJECTABLE ANTIBIOTIC use in sows in the farrowing area.

Check here ❍ if no injectable antibiotics were given to sows in this farrowing area during the farrowing period.

IMPORTANT: If for one of the active ingredients listed there is more than one type of use, then fill in a **new line** for each type of use.
For example if the same antimicrobial(s) are given:

- **At different ages (e.g. at farrowing and again later in the farrowing period)**
- **For different disease indications**

**Product Name and Concentration** (mg/ml)	**Name of Active Antimicrobial Ingredient(s)** **See the Appendix for assistance*	**Volume Given to Each Sow per Day** (mls)	**Number of Days Given**	**Average Weight at Treatment** (kgs)	**Primary Reason For Medication Use** Choose **only one** primary reason *Disease prevention OR Disease treatment*		**Percent of Sows Exposed**: Estimate the % of sows in this farrowing area that were medicated for the number of days indicated.
					**If Disease Prevention** (*Check all that apply*)	**If Disease Treatment** (*Check all that apply*)	
**Name:**<br><br>**mg/ml:**		mls	days	kgs	❑ Respiratory disease<br>❑ Enteric disease<br>❑ Lameness<br>❑ Other (Specify):	❑ Respiratory disease<br>❑ Enteric disease<br>❑ Lameness<br>❑ Other (Specify):	
**Name:**<br><br>**mg/ml:**		mls	days	kgs	❑ Respiratory disease<br>❑ Enteric disease<br>❑ Lameness<br>❑ Other (Specify):	❑ Respiratory disease<br>❑ Enteric disease<br>❑ Lameness<br>❑ Other (Specify):	
**Name:**<br><br>**mg/ml:**		mls	days	kgs	❑ Respiratory disease<br>❑ Enteric disease<br>❑ Lameness<br>❑ Other (Specify):	❑ Respiratory disease<br>❑ Enteric disease<br>❑ Lameness<br>❑ Other (Specify):	
**Name:**<br><br>**mg/ml:**		mls	days	kgs	❑ Respiratory disease<br>❑ Enteric disease<br>❑ Lameness<br>❑ Other (Specify):	❑ Respiratory disease<br>❑ Enteric disease<br>❑ Lameness<br>❑ Other (Specify):	

**Herd ID Code:**
**Vet. ID Code:**

SOW HEALTH INFORMATION

19. HEALTH STATUS of the sows in this farrowing unit.

Disease/ Syndrome	Sow Herd Disease Status<br>Confirmed status is based on Laboratory diagnosis					Were antibiotics used to *prevent or treat* this condition in sows?			Are the sows vaccinated against this disease?		
	Don't Know	Likely Neg.	Confirmed Neg.	Likely Positive	Confirmed Positive	Yes	Don't know	No	Yes	Don't Know	No
A. PRRS[1]	❍	❍	❍	❍	❍	❑	❑	❑	❍	❍	❍
B. *Mycoplasma*	❍	❍	❍	❍	❍	❑	❑	❑	❍	❍	❍
C. APP[2]	❍	❍	❍	❍	❍	❑	❑	❑	❍	❍	❍
D. Influenza	❍	❍	❍	❍	❍	❑	❑	❑	❍	❍	❍
E. Circovirus Assoc. Dis. (PCVAD)	❍	❍	❍	❍	❍	❑	❑	❑	❍	❍	❍
F. *Salmonella*	❍	❍	❍	❍	❍	❑	❑	❑	❍	❍	❍
G. *E. coli*	❍	❍	❍	❍	❍	❑	❑	❑	❍	❍	❍
H. *Erysipelas*	❍	❍	❍	❍	❍	❑	❑	❑	❍	❍	❍
I. *Streptococcus suis*	❍	❍	❍	❍	❍	❑	❑	❑	❍	❍	❍
J. Ileitis (*Lawsonia*)	❍	❍	❍	❍	❍	❑	❑	❑	❍	❍	❍
K. *H. parasuis*	❍	❍	❍	❍	❍	❑	❑	❑	❍	❍	❍
L. PED[3]	❍	❍	❍	❍	❍	❑	❑	❑	❍	❍	❍
M. Other Specify:	❍	❍	❍	❍	❍	❑	❑	❑	❍	❍	❍

[1] PRRS: *Porcine Reproductive & Respiratory Syndrome* [2] APP: *Actinobacillus pleuropneumonia*
[3] PED: *Porcine Epidemic Diarrhea*

FARM SWINE QUESTIONNAIRE	Herd ID Code:
CIPARS.FS.2017<br>**FARROWING PERIOD**	**Vet. ID Code:**

NURSING PIG HEALTH INFORMATION

20. **HEALTH STATUS of the nursing piglets in this farrowing unit.**

Disease/ Syndrome	Piglet Disease Status<br>Confirmed status is based on Laboratory diagnosis					Were antibiotics used to *prevent or treat* this condition in nursing pigs?			Are the nursing pigs vaccinated against this disease?		
	Don't Know	Likely Neg.	Confirmed Neg.	Likely Positive	Confirmed Positive	Yes	Don't know	No	Yes	Don't Know	No
A. PRRS[1]	❍	❍	❍	❍	❍	❑	❑	❑	❍	❍	❍
B. *Mycoplasma*	❍	❍	❍	❍	❍	❑	❑	❑	❍	❍	❍
C. APP[2]	❍	❍	❍	❍	❍	❑	❑	❑	❍	❍	❍
D. Influenza	❍	❍	❍	❍	❍	❑	❑	❑	❍	❍	❍
E. Circovirus Assoc. Dis. (PCVAD)	❍	❍	❍	❍	❍	❑	❑	❑	❍	❍	❍
F. *Salmonella*	❍	❍	❍	❍	❍	❑	❑	❑	❍	❍	❍
G. *E. coli*	❍	❍	❍	❍	❍	❑	❑	❑	❍	❍	❍
H. *Erysipelas*	❍	❍	❍	❍	❍	❑	❑	❑	❍	❍	❍
I. *Streptococcus suis*	❍	❍	❍	❍	❍	❑	❑	❑	❍	❍	❍
J. Ileitis (*Lawsonia*)	❍	❍	❍	❍	❍	❑	❑	❑	❍	❍	❍
K. *H. parasuis*	❍	❍	❍	❍	❍	❑	❑	❑	❍	❍	❍
L. PED[3]	❍	❍	❍	❍	❍	❑	❑	❑	❍	❍	❍
M. Other Specify:	❍	❍	❍	❍	❍	❑	❑	❑	❍	❍	❍

[1] PRRS: *Porcine Reproductive & Respiratory Syndrome* [2] APP: *Actinobacillus pleuropneumonia*
[3] PED: *Porcine Epidemic Diarrhea*

21. If an electronic application was developed that could be used to record and submit on-farm antimicrobial use information on your cell phone or tablet, would you be interested in using it?

❍ Yes ❍ No ❍ Don't Know

Thank you!

APPENDIX: VETERINARY ANTIMICROBIAL PRODUCTS*

*Sources: Compendium of Veterinary Products -Canadian Version; Compendium of Medicating Ingredients Brochure

A. In Feed Medications

Product Name (Brand Name)	Active Ingredient(s)
Surmax 200 Premix	Avilamycin
Albac 110 Zinc Bacitracin	Bacitracin
Bacitracin MD	
Bmd 110g	
Baciferm-PB-50	Bacitracin, Penicillin G
Flavomycin	Bambermycin
Aureomycin 50, 110, 220g	Chlortetracycline
Chlor 50, 100g Granular Premix	
Co-Op Aureomycin Vitamin Premix Crumbles	
Deracin 22% Granular Premix	
Aureo S-P 250g	Chlortetracycline, Penicillin G, Sulfamethazine
Aureomix 625g	
Chlor 250g Granular Premix	
Super Chlor 250g Granular Premix	
Super Chlorosol 250 Premix	
Lincomix 44, 110g Premix	Lincomycin
Lincomycin 44, 100g Premix	
Lincomycin Spectinomycin 4.4% G Premix	Linocmycin, Spectinomycin
L-S 20 Premix	
Monteban 70, 100	Narasin (anti-coccidial)
Oxy 110, 220, 440	Oxytetracycline
Oxy Tetra Forte	
Oxy Tetra-A	
Oxysol 220, 440	
Oxytetracycline 50, 100, 200 Granular Premix	
Terramycin -50, 100, 200 Premix	
Posistac 6% Premix	Salinomycin (anti-coccidial)
Coxistac 6% Premix	
Pulmotil	Tilmicosin
Tilmovet	
Tiamulin 1.78% Premix	Tiamulin
Tiamulin Hf 10% Premix	
Denagard 10% GF Premix	
Denagard Medicated Premix	
Tylan 10, 40, 100 Premix	Tylosin
Tylosin 10, 40 Premix	
Pharmasin 100 Premix	
Tylan 50/Sulfa G Premix	Tylosin,
Aivlosin 17% Premix	Tylvalosin
Virginiamycin 44 Premix	Virginiamycin
Stafac 22, 44, 500	

Veterinary Antimicrobial Products continued.

B. In Water/Oral Medications

Product Name (Brand Name)	Active Ingredient(s)
Amoxicillin SP	Amoxicillin
Paracillin SP	
Apralan	Apramycin
Lincomix SP	Lincomycin
Lincomycin Soluble Powder	
Lincomycin-Spectinomycin 100 SP	Lincomycin, Spectinomycin
Linco-SPectin 100 SP	
Neomycin 325	Neomycin
Neomed 325	
Neomycin SP	
Scour Solution	
Neooxytet SP	Neomycin, Oxytetracycline
Neotet Soluble Concentrate	
Neox	
Neo-Tetramed	Neomycin, Tetracycline
Neo-Chlor	
Calf Scour Bolus, Super Calf Scour Bolus	Neomycin, Sulfaguanidine, Sulfathiazole
Pig Zest	Neomycin, Streptomycin
Oxy-Tetra A	Oxytetracycline
Oxy 250, 1000	
Oxy Tetra Forte	
Oxysol 62.5, 1000	
Oxytet 1000 SP	
Oxytetracycline Hcl SP 1000	
Pencillin G Potassium USP SP	Penicillin G
Pot-Pen	
Booster P S Conc	Penicillin G, Streptomycin
Superbooster	
Vibiomed Booster	
SPectam Oral Solution	Spectinomycin
SPectam Scour Halt	
Sulfamethazine Bolus	Sulfamethazine
Sodium Sulfamethanzine Sol (12.5%, 25%)	
Sulfa 25% Solution	
Sulfamethazine 25% Solution	
Powder 21	Sulfamethazine, Sulfathiazole
2 Sulfamed	
Sulfa 2 Soluble Powder	
Sulfa Mt	
3- Sulvit	Sulfamethazine, Sulfathiazole, Sulfamerazine
Sulfavite	
Sulmed Plus	
Neutral Sulfa	Sulfamethazine, Sulfathiazole, Sulfapyridine
Triple Sulfa Bolus	Sulfamethazine, Sulfathiazole, Sulfanilamide
Sulectim 100	Sulfamerazine, Sulfathiazole
Onycin	Tetracycline
Tetra 55, 250, 1000	
Tetracycline 250, 1000	
Tetracycline Hydrochloride	
Tetramed	
Denagard 12.5% Liquid Concentrate	Tiamulin
Tiamulin SP	
Tylan SP	
Aivlosin Water Soluble Granules	Tyvalosin
Baycox	Toltrazuril (anti-coccidial)

Veterinary Antimicrobial Products continued.

C. Injectable Medications

Product Name (Brand Name)	Active Ingredient(s)
Polyflex	Ampicillin
Depocillin	Procaine Penicillin G
Hi-Pencin 200	
Pen G Injection	
Pen Vet 300	
Penpro	
Proc Pen LA	
Procaine Pencillin G	
Procillin	
Duplocillin LA	Procaine Penicillin G, Benzathine Pencillin G
Ceftiocyl	Ceftiofur
Ceftiofur Sodium for Injection	
Eficur	
Excenel, Excenel RTU EZ, Excenel RTU	
Excede 100	
Baytril 100	Enrofloxacin
Nuflor	Florifenicol
Gentocin	Gentamicin
Lincomed 100	Lincomycin
Lincomix 100	
Alamycin	Oxytetracycline
Bio-mycin 200	
Cyclosol 200	
Liquamycin LA 200	
Noromycin LA, Noromycin LA 300	
Noromycin LP	
Oxymycin LA, LP	
Oxytetracycline 100 LP	
Oxytetramycin 100	
Oxyvet 100 LP	
Oxyvet 200 LA	
Borgal	Sulfadoxine, Trimethroprim
Dofatrim-Ject	
Norovet TMPS	
Trimidox	
Trivetrin	
Denagard Injection	Tiamulin
Draxxin, Draxxin 25	Tulathromycin
Tylan 200	Tylosin
