# Supplementary material for: Antimicrobial use in lactating sows, piglets, nursery, and grower-finisher pigs on swine farms in Ontario, Canada during 2017 and 2018
Source: Porcine Health Manag. 2022 Apr 28;8:17. doi: 10.1186/s40813-022-00259-w (PMC9047262; doi:10.1186/s40813-022-00259-w)
Supplement: Supplementary file 2 — Additional file 2. The nursery questionnaire used to collect information on antimicrobial use, biosecurity practices, health status, and animal numbers in Ontario nursery pig herds, May 2017–April 2018. An English copy of the farrowing questionnaire used to collect data from participating nursery pig herds in Ontario, Canada (May 2017–April 2018). [file 40813_2022_259_MOESM2_ESM.pdf]

FARM SWINE QUESTIONNAIRE
CIPARS.FS.2017<br>NURSERY PERIOD

Herd ID Code:
Vet. ID Code:

CANADIAN INTEGRATED PROGRAM FOR ANTIMICROBIAL RESISTANCE SURVEILLANCE (CIPARS)[1]

NURSING/NURSERY PIG ANTIMICROBIAL USE RESEARCH PROJECT

QUESTIONNAIRE

NURSERY OPERATIONS

INSTRUCTIONS:

PLEASE READ CAREFULLY

- ☐ If you do not know how to answer any question **please feel free to contact:**

  Louise Bellai: 519-826-2348

- ☐ Complete this questionnaire based on a **single nursery period**.

- ☐ **PLEASE SAMPLE BEFORE A SUBSTANTIAL NUMBER OF PIGS FROM THE GROUP HAVE BEEN TRANSFERRED TO THE GROWER-FINISHER UNIT**

- ☐ **Pigs sampled today should:**
  - Be between 9 to 12 weeks of age;
  - Be between 20 and 25 kgs of body weight
  - Not be from slush (sick) or cull pens.

- ☐ **The nursery area must be defined in Question 2. All subsequent questions must be answered on this basis (barn or room).**

- ☐ For your reference, an **appendix** listing some of the veterinary antimicrobial products available for use in swine can be found at the end of the questionnaire.

- ☐ Enter the **Herd ID** and **Vet ID** codes in the boxes on the top right corner of **each page**.

- ☐ **Please answer all questions**
  - If the producer does not know the answer, indicate "D/K" as the response
  - If a question is not applicable to the herd, indicate "N/A" as the response.

- ☐ This questionnaire is in triplicate
  - **White** copy: Send to CIPARS in the express post envelope provided;
  - **Yellow** copy: Retained by the herd veterinarian;
  - **Pink** copy: Retained by the owner of the barn.

[1] CIPARS – A national program that monitors antimicrobial use and resistance.

Herd ID Code:
Vet. ID Code:

REPORTING INFORMATION

1. **Date** this questionnaire was completed and samples collected: ______ / ______ / ______ (Month / Day / Year)

2. **Nursery Area***: All data, including pig inventory for this questionnaire are provided by the:

❑ Room ❑ Barn
(*Check only one please*)

***Questions 9 through 17 must all refer to the same nursery group in the nursery area.**

For this questionnaire, **nursery group** refers to the pigs that were weaned and moved into the nursery together as a group, within a period of 7 days.

3. **Dates and samples that these data pertain to:**

Continuous flow operations: (*The entire room/barn* ***is not*** *emptied of pigs at the end of each nursery period; at least one or more pig(s) remain in the room/barn*.). The start date would be today's date minus the average nursery period, as indicated in question 9A.

All-in-all-out operations: (*The entire room/barn* ***is*** *emptied of pigs at the end of each nursery period,* ***no pigs*** *remain in the room/barn*.) The start date would be the day that the pigs sampled today were weaned and transferred to the nursery. The projected end date is the date when it is anticipated that all of the pigs from the same nursery group as the pigs sampled today will be transferred to the grower-finisher unit, as indicated in question 9A.

A. Start date: ______ / ______ / ______ (Month / Day / Year)

B. End date for **Continuous Flow** operations is today's date: ______ / ______ / ______ (Month / Day / Year)

C. Projected end date for **All-In-All-Out** operations: ______ / ______ / ______ (Month / Day / Year)

White copy: Public Health Agency; **Yellow copy**: Herd Veterinarian; **Pink copy**: Producer

Herd ID Code:

Vet. ID Code:

SAMPLE INFORMATION

D. Please complete the table below for the six nursery pens sampled today:

***REMINDER**

Pigs sampled today should:

- Be between 9 to 12 weeks of age;
- Be between 20 and 25 kgs of body weight;
- Not be from slush (sick) or cull pens.
- The six (6) pens sampled today should be located in the same nursery area (as defined in Question 2).

Sample ID Code	Minimum Age	Maximum Age	Minimum Weight	Maximum Weight
	Weeks	Weeks	Circle: Kgs	or Lbs
	Weeks	Weeks		
	Weeks	Weeks		
	Weeks	Weeks		
	Weeks	Weeks		
	Weeks	Weeks		

Herd ID Code:
Vet. ID Code:

GENERAL HERD AND SITE INFORMATION

4. Herd Information:

A. Is the nursery operation sampled today: ❍ Independent ❍ Part of a production group ❍ Don't Know

B. Do you have a farrowing operation?: ❍ Yes ❍ No

If ***yes:***

i. Is it: ❍ On-site ❍ Off-Site

ii. Participating in this project: ❍ Yes ❍ No

If yes, please provide the herd ID: ____________________

C. Do you have a grower-finisher operation?: ❍ Yes ❍ No

If ***yes***:

i. Is it: ❍ On-site ❍ Off-Site

ii. Is it a CIPARS herd: ❍ Yes ❍ No

If yes, please provide the herd ID: ____________________

5. What is the total number of sows in the herd(s) supplying this nursery operation?

________________ Sows

6. Are the pigs sampled today part of a “Raised without Antibiotics” (RWA) production system? ❍ Yes ❍ No ❍ Don’t Know

A. If RWA, what was the last date when antibiotics were used in nursery pigs in this system? ____ / ____ / ____ (Month / Day / Year)

B. If RWA, are antibiotic treatments permitted in boars and gestating sows? ❍ Yes ❍ No ❍ Don’t Know

C. If RWA, are antibiotic treatments permitted in lactating sows? ❍ Yes ❍ No ❍ Don’t Know

7. Level of Biosecurity:

Boots provided by farm:	❍Yes	❍No	
Coveralls provided by farm:	❍Yes	❍No	
Boot dip:	❍Yes	❍No	
Biosecurity Sign:	❍Yes	❍No	
Danish Entry:	❍Yes	❍No	
Locked Doors:	❍Yes	❍No	
Restricting visitors:	❍Yes	❍No	
Shower:	❍Yes	❍No	
Downtime*:	❍Yes	❍No	Hours of downtime: ________
Other:	❍Yes	❍No	Specify: ________

***Note:** Downtime here refers to the requirement for visitors and personnel to refrain from visiting the farm for a certain length of time after contact with other pigs/swine farms.

8. Number of pig farms located within two kilometres of this site: ________ Farm(s)

NURSERY PIG INFORMATION

ATTENTION:

- All questionnaire data, including pig numbers must correspond to the same nursery group and nursery area, as defined in question 2.
- **These estimates are critical to our analysis.**

---

9. Nursery Pig Numbers

CONTINUOUS FLOW OPERATIONS (*For All-In-All-Out operations please proceed to the next page*)

A. **Nursery Period**: What is the average number of weeks that pigs are in the nursery phase of production? ____________ Weeks

The following questions (9B through 9F) refer to the timeframe defined by today's date minus the average number of weeks in the nursery, as indicated above in 9A

B. **Pig Inflow**: Estimate the number of pigs that entered this nursery area during the number of weeks specified above in 9A. **This entry should equal the number of pigs entering the nursery area each week multiplied by the number of weeks specified in 9A.** ____________ Pigs

C. **Post-weaning mortality:** Estimate the number or percent of mortalities in this nursery area, during the number of weeks specified in 9A. ____________ Percent

OR

____________ Pigs

D. **Pigs Transferred**: Estimate the number of pigs transferred to the grower-finisher unit, from this nursery room or barn (see Q.2), during the number of weeks specified above in 9A. **This entry should equal the number of pigs transferred to the grow-finish phase of production each week multiplied by the number of weeks specified in 9A.** ____________ Pigs

E. **Pigs Sold:** Estimate the number of pigs sold as lightweight or BBQ pigs during the number of weeks specified in 9A. ____________ Pigs

F. **Pigs today**: Estimate the number of pigs that are in this nursery area today. ____________ Pigs

G. What is the **nursery pig capacity** of this barn? ____________ Pigs

9. Nursery Pig Numbers

ALL-IN-ALL-OUT OPERATIONS

A. **Starting Inventory**: Estimate the number of pigs that entered the nursery phase of production with the pigs sampled today. __________ Pigs

B. **Post-weaning mortality**: Estimate the number or percent of mortalities in this nursery area (Q. 2). __________ Percent OR __________ Pigs

C. **Pigs Sold:** Estimate the number of pigs sold as lightweight or BBQ pigs in this nursery area. __________ Pigs

D. **Pigs today***: Estimate the number of pigs in this nursery area that are in the room/barn today. __________ Pigs*

* **Note:** The answer for 9D should equal the # of pigs in 9A minus the mortality (9B) and the pigs sold (9C).

E. What is the nursery pig capacity of this barn? __________ Pigs

Herd ID Code:
Vet. ID Code:

Attention: The following applies to the feed, injectable, water and oral antimicrobial information tables for both the nursing piglets and the sows:

For both **Continuous Flow** and **All-In-All-Out** operations these data pertain to the nursery group that was specified in Questions 3 and 9, and the nursery area specified in Question 2.
Disease Treatment: an antimicrobial was started because a pig in the barn was suffering from a disease or condition of concern.

Disease Prevention: an antimicrobial was started but no pig was sick at the time.

Growth Promotion: an antimicrobial was used to improve growth or feed efficiency only.

Pulsed medication: a repetitive short-term medication protocol, e.g. 3 days of medication followed by 7 days without medication, repeated 3 times.

10. **Age at weaning:** What was the average weaning age, in days, of the pigs sampled today? ____________ Days

11. **LIST ALL THE RATIONS used to feed nursery pigs during the nursery period that was specified in questions 3 and 9.** A nursery period is the total number of weeks that pigs are in the nursery unit. In most barns this would be a 6 to 8 week period.

Attention: These data are critical to our analysis

Ration Name	Start Weight of Pigs	End Weight of Pigs	Average # Weeks Fed per Nursery Period
	Circle: Kgs	or Lbs	
**NOTE:** THIS TOTAL SHOULD EQUAL THE NUMBER OF WEEKS THAT IT TAKES THE AVERAGE PIG TO GO FROM THE START OF THE NURSERY PERIOD (WEANING) TO TRANSFER TO THE GROW-FINISH BARN ⇨		**Total # of weeks that pigs are in the nursery unit:**	

12. IN THE TABLE BELOW COMPLETE ONE LINE FOR EACH RATION LISTED ABOVE INCLUDING NON-MEDICATED RATIONS.

NOTE: The ration names provided in the table above (Question 11**) MUST correspond** to the ration names used in the table below**.**

IMPORTANT: Nursery pig feed information provided below should be for the same nursery area indicated in Question 2 (Reporting level: Barn or Room) and for the nursery period indicated in Question 3.

If for one of the rations listed in Question 11 there is **ANY** change in **MEDICATION** then fill in a new line for each change e.g. medicated to non-medicated, change in inclusion rates, change in drug incorporated. You do not need to start a new line if there is a change in ration formulation from a nutrient perspective. A new line is only needed for medication changes. **Additional space** is available over the **next two pages**.

Attention: These data are critical to our analysis. Please fill in all of the information requested.

Ration Name (from Question 11)	Medicated?	**Primary Reason For Medication Use** *Choose **only one** primary reason by checking "Yes": Growth promotion OR Disease prevention OR Treatment*			Name of Active Antimicrobial Ingredient(s) **See the Appendix for assistance*	Grams of Active Ingredient per Tonne (g/tonne)	**Percent of Pigs Fed**: Estimate the % of nursery pigs in this nursery area that were fed each ration for number of weeks indicated in Q.11.
		**Growth Promotion**	**Disease Prevention** (If Yes, *check all disease checkboxes that apply)*	**Disease Treatment** (If Yes, *check all disease checkboxes that apply)*			
	❍ Yes<br>❍ No<br>❑ Pulsed<br>Total Days: ______	❍Yes	❍Yes:<br>❑ Respiratory disease<br>❑ Enteric disease<br>❑ Lameness<br>❑ Other (Specify):	❍Yes:<br>❑ Respiratory disease<br>❑ Enteric disease<br>❑ Lameness<br>❑ Other (Specify):		g/tonne	
	❍ Yes<br>❍ No<br>❑ Pulsed<br>Total Days: ______	❍Yes	❍Yes:<br>❑ Respiratory disease<br>❑ Enteric disease<br>❑ Lameness<br>❑ Other (Specify):	❍Yes:<br>❑ Respiratory disease<br>❑ Enteric disease<br>❑ Lameness<br>❑ Other (Specify):		g/tonne	

This *feed information table* is continued on the following pages

Nursery Pig Feed information table continued.

**Ration Name** (from Question 11)	**Medicated?**	**Primary Reason For Medication Use** *Choose **only one** primary reason by checking "Yes": Growth promotion OR Disease prevention OR Treatment*			**Name of Active Antimicrobial Ingredient(s)** **See the Appendix for assistance*	**Grams of Active Ingredient per Tonne** (g/tonne)	**Percent of Pigs Fed**: Estimate the % of nursery pigs in this nursery area that were fed each ration for number of weeks indicated in Q.11.
		**Growth Promotion**	**Disease Prevention** (If Yes, *check all disease checkboxes that apply)*	**Disease Treatment** (If Yes, *check all disease checkboxes that apply)*			
	❍ Yes<br>❍ No<br>❑ Pulsed<br>Total Days: ______	❍Yes	❍Yes:<br>❑ Respiratory disease<br>❑ Enteric disease<br>❑ Lameness<br>❑ Other (Specify):	❍Yes:<br>❑ Respiratory disease<br>❑ Enteric disease<br>❑ Lameness<br>❑ Other (Specify):		g/tonne	
	❍ Yes<br>❍ No<br>❑ Pulsed<br>Total Days: ______	❍Yes	❍Yes:<br>❑ Respiratory disease<br>❑ Enteric disease<br>❑ Lameness<br>❑ Other (Specify):	❍Yes:<br>❑ Respiratory disease<br>❑ Enteric disease<br>❑ Lameness<br>❑ Other (Specify):		g/tonne	
	❍ Yes<br>❍ No<br>❑ Pulsed<br>Total Days: ______	❍Yes	❍Yes:<br>❑ Respiratory disease<br>❑ Enteric disease<br>❑ Lameness<br>❑ Other (Specify):	❍Yes:<br>❑ Respiratory disease<br>❑ Enteric disease<br>❑ Lameness<br>❑ Other (Specify):		g/tonne	
	❍ Yes<br>❍ No<br>❑ Pulsed<br>Total Days: ______	❍Yes	❍Yes:<br>❑ Respiratory disease<br>❑ Enteric disease<br>❑ Lameness<br>❑ Other (Specify):	❍Yes:<br>❑ Respiratory disease<br>❑ Enteric disease<br>❑ Lameness<br>❑ Other (Specify):		g/tonne	

This *feed information table* is continued on the following pages

Nursery Pig Feed information table continued.

**Ration Name** (from Question 11)	**Medicated?**	**<u>Primary</u> Reason For Medication Use** *Choose **only one** primary reason by checking "Yes": Growth promotion OR Disease prevention OR Treatment*			**Name of Active Antimicrobial Ingredient(s)** **See the Appendix for assistance*	**Grams of Active Ingredient per Tonne** (g/tonne)	**Percent of Pigs Fed**: Estimate the % of nursery pigs in this nursery area that were fed each ration for number of weeks indicated in Q.11.
		**Growth Promotion**	**Disease Prevention** (If Yes, *check all disease checkboxes that apply)*	**Disease Treatment** (If Yes, *check all disease checkboxes that apply)*			
	❍ Yes<br>❍ No<br>❑ Pulsed<br>Total Days: ______	❍Yes	❍Yes:<br>❑ Respiratory disease<br>❑ Enteric disease<br>❑ Lameness<br>❑ Other (Specify):	❍Yes:<br>❑ Respiratory disease<br>❑ Enteric disease<br>❑ Lameness<br>❑ Other (Specify):		g/tonne	
	❍ Yes<br>❍ No<br>❑ Pulsed<br>Total Days: ______	❍Yes	❍Yes:<br>❑ Respiratory disease<br>❑ Enteric disease<br>❑ Lameness<br>❑ Other (Specify):	❍Yes:<br>❑ Respiratory disease<br>❑ Enteric disease<br>❑ Lameness<br>❑ Other (Specify):		g/tonne	
	❍ Yes<br>❍ No<br>❑ Pulsed<br>Total Days: ______	❍Yes	❍Yes:<br>❑ Respiratory disease<br>❑ Enteric disease<br>❑ Lameness<br>❑ Other (Specify):	❍Yes:<br>❑ Respiratory disease<br>❑ Enteric disease<br>❑ Lameness<br>❑ Other (Specify):		g/tonne	

13. Does this nursery use an extra-physiological level of zinc in feed? ❍ Yes ❍ No

If <u>yes</u>, how many parts per million (ppm) of zinc are added? ________________ ppm

FARM SWINE QUESTIONNAIRE

CIPARS.FS.2017
NURSERY PERIOD

Herd ID Code:
Vet. ID Code:

14. ANTIBIOTICS ADMINISTERED IN WATER in nursery pigs. List continued on following page.

Check here ❍ if no antibiotics were given in water to nursery pigs during this period.

IMPORTANT: If for one of the active ingredients listed there is more than one type of use, then fill in a **new line** for each type of use.
For example if the same antimicrobial(s) are given:

- **At different ages (e.g. at weaning and again later in the nursery period)**
- **For different disease indications**

**Product Name and Concentration** (mg/g, mg/pkg, IU/g, other) **Please indicate units*	**Name of Active Antimicrobial Ingredient(s)** **See the Appendix for assistance*	**Grams of Active Ingredient per Litre of Water** (g/L)	**Number of Days Given**	**Average Age at Start of Treatment** (weeks)	**Average Weight at Start of Treatment** (kgs)	**Primary Reason For Medication Use** Choose **only one** primary reason *Disease prevention OR Disease treatment*		**Percent of Pigs Exposed**: Estimate the % of nursery pigs in this nursery area that were medicated for the number of days indicated.
						**If Disease Prevention** *(Check all that apply)*	**If Disease Treatment** *(Check all that apply)*	
**Name:**<br><br>**Concentration:** ❍ mg/g ❍ mg/pkg ❍ IU/g ❍ Other (Specify): ________		g/L	days	weeks	kgs	❑ Respiratory disease<br>❑ Enteric disease<br>❑ Lameness<br>❑ Other (Specify):	❑ Respiratory disease<br>❑ Enteric disease<br>❑ Lameness<br>❑ Other (Specify):	
**Name:**<br><br>**Concentration:** ❍ mg/g ❍ mg/pkg ❍ IU/g ❍ Other (Specify): ________		g/L	days	weeks	kgs	❑ Respiratory disease<br>❑ Enteric disease<br>❑ Lameness<br>❑ Other (Specify):	❑ Respiratory disease<br>❑ Enteric disease<br>❑ Lameness<br>❑ Other (Specify):	

This *Medicated Water in Nursery pigs* table is continued on the following page.

FARM SWINE QUESTIONNAIRE

CIPARS.FS.2017
NURSERY PERIOD

**Herd ID Code:**
**Vet. ID Code:**

Medicated Water in Nursery Pigs table continued

**Product Name and Concentration** (mg/g, mg/pkg, IU/g, other)<br><br>**Please indicate units*	**Name of Active Antimicrobial Ingredient(s)**<br><br>**See the Appendix for assistance*	**Grams of Active Ingredient per Litre of Water** (g/L)	**Number of Days Given**	**Average Age at Start of Treatment** (weeks)	**Average Weight at Start of Treatment** (kgs)	**<u>Primary</u> Reason For Medication Use**<br>Choose **only one** primary reason *Disease prevention OR Disease treatment*<br>**If Disease <u>Prevention</u>** *(Check all that apply)*	**If Disease <u>Treatment</u>** *(Check all that apply)*	**Percent of Pigs Exposed**: Estimate the % of nursery pigs in this nursery area that were medicated for the number of days indicated.
**Name:**<br><br>**Concentration:** ❍ mg/g ❍ mg/pkg ❍ IU/g ❍ Other (Specify): ________<br>____________		g/L	days	weeks	kgs	❑ Respiratory disease<br>❑ Enteric disease<br>❑ Lameness<br>❑ Other (Specify):	❑ Respiratory disease<br>❑ Enteric disease<br>❑ Lameness<br>❑ Other (Specify):	
**Name:**<br><br>**Concentration:** ❍ mg/g ❍ mg/pkg ❍ IU/g ❍ Other (Specify): ________<br>____________		g/L	days	weeks	kgs	❑ Respiratory disease<br>❑ Enteric disease<br>❑ Lameness<br>❑ Other (Specify):	❑ Respiratory disease<br>❑ Enteric disease<br>❑ Lameness<br>❑ Other (Specify):	
**Name:**<br><br>**Concentration:** ❍ mg/g ❍ mg/pkg ❍ IU/g ❍ Other (Specify): ________<br>____________		g/L	days	weeks	kgs	❑ Respiratory disease<br>❑ Enteric disease<br>❑ Lameness<br>❑ Other (Specify):	❑ Respiratory disease<br>❑ Enteric disease<br>❑ Lameness<br>❑ Other (Specify):	

Herd ID Code:

Vet. ID Code:

15. Injectable Antibiotic Use in nursery pigs. List continued on the following page.

Check here ❍ if no injectable antibiotics were given to nursery pigs during this period.

IMPORTANT: If for one of the active ingredients listed there is more than one type of use, then fill in a **new line** for each type of use.
For example if the same antimicrobial(s) are given:

- **At different ages (e.g. at weaning and again later in the nursery period)**
- **For different disease indications**

**Product Name and Concentration** (mg/ml)	**Name of Active Antimicrobial Ingredient(s)** **See the Appendix for assistance*	**Volume Given to Each Pig per Day** (mls)	**Number of Days Given**	**Average Age at Start of Treatment** (weeks)	**Average Weight at Start of treatment** (kgs)	**Primary Reason For Medication Use** Choose **only one** primary reason *Disease prevention OR Disease treatment*		**Percent of Pigs Exposed**: Estimate the % of nursery pigs in this nursery area that were medicated for the number of days indicated.
						**If Disease Prevention** *(Check all that apply)*	**If Disease Treatment** *(Check all that apply)*	
**Name:**<br>**mg/ml:**		mls	days	weeks	kgs	❑ Respiratory disease<br>❑ Enteric disease<br>❑ Lameness<br>❑ Other (Specify):	❑ Respiratory disease<br>❑ Enteric disease<br>❑ Lameness<br>❑ Other (Specify):	
**Name:**<br>**mg/ml:**		mls	days	weeks	kgs	❑ Respiratory disease<br>❑ Enteric disease<br>❑ Lameness<br>❑ Other (Specify):	❑ Respiratory disease<br>❑ Enteric disease<br>❑ Lameness<br>❑ Other (Specify):	
**Name:**<br>**mg/ml:**		mls	days	weeks	kgs	❑ Respiratory disease<br>❑ Enteric disease<br>❑ Lameness<br>❑ Other (Specify):	❑ Respiratory disease<br>❑ Enteric disease<br>❑ Lameness<br>❑ Other (Specify):	

This *Injectable Antibiotics in Nursery Pigs table* is continued on the following page.

**FARM SWINE QUESTIONNAIRE**
**CIPARS.FS.2017**<br>**NURSERY PERIOD**

**Herd ID Code:**
**Vet. ID Code:**

Injectable Antibiotics in Nursery Pigs table continued

**Product Name and Concentration** (mg/ml)	**Name of Active Antimicrobial Ingredient(s)**<br>**See the Appendix for assistance*	**Volume Given to Each Pig per Day** (mls)	**Number of Days Given**	**Average Age at Start of Treatment** (weeks)	**Average Weight at Start of treatment** (kgs)	**Primary Reason For Medication Use** Choose **only one** primary reason *Disease prevention OR Disease treatment*<br>**If Disease Prevention** (*Check all that apply)*	**If Disease Treatment** (*Check all that apply)*	**Percent of Pigs Exposed**: Estimate the % of nursery pigs in this nursery area that were medicated for the number of days indicated.
**Name:**<br>**mg/ml:**		mls	days	weeks	kgs	❑ Respiratory disease<br>❑ Enteric disease<br>❑ Lameness<br>❑ Other (Specify):	❑ Respiratory disease<br>❑ Enteric disease<br>❑ Lameness<br>❑ Other (Specify):	
**Name:**<br>**mg/ml:**		mls	days	weeks	kgs	❑ Respiratory disease<br>❑ Enteric disease<br>❑ Lameness<br>❑ Other (Specify):	❑ Respiratory disease<br>❑ Enteric disease<br>❑ Lameness<br>❑ Other (Specify):	
**Name:**<br>**mg/ml:**		mls	days	weeks	kgs	❑ Respiratory disease<br>❑ Enteric disease<br>❑ Lameness<br>❑ Other (Specify):	❑ Respiratory disease<br>❑ Enteric disease<br>❑ Lameness<br>❑ Other (Specify):	
**Name:**<br>**mg/ml:**		mls	days	weeks	kgs	❑ Respiratory disease<br>❑ Enteric disease<br>❑ Lameness<br>❑ Other (Specify):	❑ Respiratory disease<br>❑ Enteric disease<br>❑ Lameness<br>❑ Other (Specify):	

NURSERY PIG HEALTH INFORMATION

16. CURRENT HEALTH STATUS of this nursery.

**Disease/ Syndrome**	**Nursery Disease Status** Confirmed status is based on Laboratory diagnosis					**Were antibiotics used to *prevent or treat* this condition in nursery pigs?**			**Are the nursery pigs vaccinated against this disease?**		
	Don't Know	Likely Neg.	Confirmed Neg.	Likely Positive	Confirmed Positive	Yes	Don't know	No	Yes	Don't Know	No
A. PRRS[1]	❍	❍	❍	❍	❍	❑	❑	❑	❍	❍	❍
B. *Mycoplasma*	❍	❍	❍	❍	❍	❑	❑	❑	❍	❍	❍
C. APP[2]	❍	❍	❍	❍	❍	❑	❑	❑	❍	❍	❍
D. Influenza	❍	❍	❍	❍	❍	❑	❑	❑	❍	❍	❍
E. Circovirus Assoc. Dis. (PCVAD)	❍	❍	❍	❍	❍	❑	❑	❑	❍	❍	❍
F. Salmonella	❍	❍	❍	❍	❍	❑	❑	❑	❍	❍	❍
G. E. coli	❍	❍	❍	❍	❍	❑	❑	❑	❍	❍	❍
H. Erysipelas	❍	❍	❍	❍	❍	❑	❑	❑	❍	❍	❍
I. Streptococcus suis	❍	❍	❍	❍	❍	❑	❑	❑	❍	❍	❍
J. Ileitis (Lawsonia)	❍	❍	❍	❍	❍	❑	❑	❑	❍	❍	❍
K. TGE[4]	❍	❍	❍	❍	❍	❑	❑	❑	❍	❍	❍
L. *H. parasuis*	❍	❍	❍	❍	❍	❑	❑	❑	❍	❍	❍
M. PED[3]	❍	❍	❍	❍	❍	❑	❑	❑	❍	❍	❍
N. Other Specify:	❍	❍	❍	❍	❍	❑	❑	❑	❍	❍	❍

[1] PRRS: *Porcine Reproductive & Respiratory Syndrome*
[2] APP: *Actinobacillus pleuropneumonia*
[3] PED: *Porcine Epidemic Diarrhea*
[4] TGE: *Transmissible Gastroenteritis*

Herd ID Code:

Vet. ID Code:

SOW HEALTH INFORMATION

17. HEALTH STATUS of the Sow herd supplying pigs to this site for this nursery period (specified in question 3 and 9).

Check here ❍ if you **do not know** the health status of the sows

Note: *If multiple sources and if it is known that at least one of the source herds was positive, then check positive.*

**Disease/ Syndrome**	**Sow Herd Disease Status** Confirmed status is based on Laboratory diagnosis					**Were antibiotics used to** *prevent or treat* **this condition in sows?**			**Are the sows vaccinated against this disease?**		
	Don't Know	Likely Neg.	Confirmed Neg.	Likely Positive	Confirmed Positive	Yes	Don't know	No	Yes	Don't Know	No
A. *PRRS*[1]	❍	❍	❍	❍	❍	❑	❑	❑	❍	❍	❍
B. *Mycoplasma*	❍	❍	❍	❍	❍	❑	❑	❑	❍	❍	❍
C. APP[2]	❍	❍	❍	❍	❍	❑	❑	❑	❍	❍	❍
D. Influenza	❍	❍	❍	❍	❍	❑	❑	❑	❍	❍	❍
E. Circovirus Assoc. Dis. (PCVAD)	❍	❍	❍	❍	❍	❑	❑	❑	❍	❍	❍
F. *Salmonella*	❍	❍	❍	❍	❍	❑	❑	❑	❍	❍	❍
G. *E. coli*	❍	❍	❍	❍	❍	❑	❑	❑	❍	❍	❍
H. *Erysipelas*	❍	❍	❍	❍	❍	❑	❑	❑	❍	❍	❍
I. *Streptococcus suis*	❍	❍	❍	❍	❍	❑	❑	❑	❍	❍	❍
J. Ileitis (*Lawsonia*)	❍	❍	❍	❍	❍	❑	❑	❑	❍	❍	❍
K. *H. parasuis*	❍	❍	❍	❍	❍	❑	❑	❑	❍	❍	❍
L. PED[3]	❍	❍	❍	❍	❍	❑	❑	❑	❍	❍	❍
M. Other Specify:	❍	❍	❍	❍	❍	❑	❑	❑	❍	❍	❍

[1] PRRS: *Porcine Reproductive & Respiratory Syndrome* [2] APP: *Actinobacillus pleuropneumonia*
[3] PED: *Porcine Epidemic Diarrhea*

18. If an electronic application was developed that could be used to record and submit on-farm antimicrobial use information on your cell phone or tablet, would you be interested in using it?

❍ Yes ❍ No ❍ Don't Know

Thank you!

Herd ID Code:
Vet. ID Code:

APPENDIX: VETERINARY ANTIMICROBIAL PRODUCTS*

*Sources: Compendium of Veterinary Products -Canadian Version; Compendium of Medicating Ingredients Brochure

A. In Feed Medications

Product Name (Brand Name)	Active Ingredient(s)
Surmax 200 Premix	Avilamycin
Albac 110 Zinc Bacitracin	Bacitracin
Bacitracin MD	
Bmd 110g	
Baciferm-PB-50	Bacitracin, Penicillin G
Flavomycin	Bambermycin
Aureomycin 50, 110, 220g	Chlortetracycline
Chlor 50, 100g Granular Premix	
Co-Op Aureomycin Vitamin Premix Crumbles	
Deracin 22% Granular Premix	
Aureo S-P 250g	Chlortetracycline, Penicillin G, Sulfamethazine
Aureomix 625g	
Chlor 250g Granular Premix	
Super Chlor 250g Granular Premix	
Super Chlorosol 250 Premix	
Lincomix 44, 110g Premix	Lincomycin
Lincomycin 44, 100g Premix	
Lincomycin SPectinomycin 4.4% G Premix	Linocmycin, Spectinomycin
L-S 20 Premix	
Monteban 70, 100	Narasin (anti-coccidial)
Oxy 110, 220, 440	Oxytetracycline
Oxy Tetra Forte	
Oxy Tetra-A	
Oxysol 220, 440	
Oxytetracycline 50, 100, 200 Granular Premix	
Terramycin -50, 100, 200 Premix	
Posistac 6% Premix	Salinomycin (anti-coccidial)
Coxistac 6% Premix	
Pulmotil	Tilmicosin
Tilmovet	
Tiamulin 1.78% Premix	Tiamulin
Tiamulin Hf 10% Premix	
Denagard 10% GF Premix	
Denagard Medicated Premix	
Tylan 10, 40, 100 Premix	Tylosin
Tylosin 10, 40 Premix	
Pharmasin 100 Premix	
Tylan 50/Sulfa G Premix	Tylosin,
Aivlosin 17% Premix	Tylvalosin
Virginiamycin 44 Premix	Virginiamycin
Stafac 22, 44, 500	

Herd ID Code:
Vet. ID Code:

Veterinary Antimicrobial Products continued.

B. In Water/Oral Medications

Product Name (Brand Name)	Active Ingredient(s)
Amoxicillin SP	Amoxicillin
Paracillin SP	
Apralan	Apramycin
Lincomix SP	Lincomycin
Lincomycin Soluble Powder	
Lincomycin-SPectinomycin 100 SP	Lincomycin, Spectinomycin
Linco-SPectin 100 SP	
Neomycin 325	Neomycin
Neomed 325	
Neomycin SP	
Scour Solution	
Neooxytet SP	Neomycin, Oxytetracycline
Neotet Soluble Concentrate	
Neox	
Neo-Tetramed	Neomycin, Tetracycline
Neo-Chlor	
Calf Scour Bolus, Super Calf Scour Bolus	Neomycin, Sulfaguanidine, Sulfathiazole
Pig Zest	Neomycin, Streptomycin
Oxy-Tetra A	Oxytetracycline
Oxy 250, 1000	
Oxy Tetra Forte	
Oxysol 62.5, 1000	
Oxytet 1000 SP	
Oxytetracycline Hcl SP 1000	
Pencillin G Potassium USP SP	Penicillin G
Pot-Pen	
Booster P S Conc	Penicillin G, Streptomycin
Superbooster	
Vibiomed Booster	
SPectam Oral Solution	Spectinomycin
SPectam Scour Halt	
Sulfamethazine Bolus	Sulfamethazine
Sodium Sulfamethanzine Sol (12.5%, 25%)	
Sulfa 25% Solution	
Sulfamethazine 25% Solution	
Powder 21	Sulfamethazine, Sulfathiazole
2 Sulfamed	
Sulfa 2 Soluble Powder	
Sulfa Mt	
3- Sulvit	Sulfamethazine, Sulfathiazole, Sulfamerazine
Sulfavite	
Sulmed Plus	
Neutral Sulfa	Sulfamethazine, Sulfathiazole, Sulfapyridine
Triple Sulfa Bolus	Sulfamethazine, Sulfathiazole, Sulfanilamide
Sulectim 100	Sulfamerazine, Sulfathiazole
Onycin	Tetracycline
Tetra 55, 250, 1000	
Tetracycline 250, 1000	
Tetracycline Hydrochloride	
Tetramed	
Denagard 12.5% Liquid Concentrate	Tiamulin
Tiamulin SP	
Tylan SP	
Aivlosin Water Soluble Granules	Tyvalosin
Baycox	Toltrazuril (anti-coccidial)

Herd ID Code:

Vet. ID Code:

Veterinary Antimicrobial Products continued.

C. Injectable Medications

Product Name (Brand Name)	Active Ingredient(s)
Polyflex	Ampicillin
Depocillin	Procaine Penicillin G
Hi-Pencin 200	
Pen G Injection	
Pen Vet 300	
Penpro	
Proc Pen LA	
Procaine Pencillin G	
Procillin	
Duplocillin LA	Procaine Penicillin G, Benzathine Penicillin G
Ceftiocyl	Ceftiofur
Ceftiofur Sodium for Injection	
Eficur	
Excenel, Excenel RTU EZ, Excenel RTU	
Excede 100	
Baytril 100	Enrofloxacin
Nuflor	Florifenicol
Gentocin	Gentamicin
Lincomed 100	Lincomycin
Lincomix 100	
Alamycin	Oxytetracycline
Bio-mycin 200	
Cyclosol 200	
Liquamycin LA 200	
Noromycin LA, Noromycin LA 300	
Noromycin LP	
Oxymycin LA, LP	
Oxytetracycline 100 LP	
Oxytetramycin 100	
Oxyvet 100 LP	
Oxyvet 200 LA	
Borgal	Sulfadoxine, Trimethroprim
Dofatrim-Ject	
Norovet TMPS	
Trimidox	
Trivetrin	
Denagard Injection	Tiamulin
Draxxin, Draxxin 25	Tulathromycin
Tylan 200	Tylosin
